# Photochemical Spin‐State Switching of an All‐Organic Molecular System with Visible Light

**DOI:** 10.1002/anie.202515144

**Published:** 2025-09-26

**Authors:** Joël Schlecht, Thomas Lohmiller, Philipp Thielert, Clara Douglas, Malte Gather, Sabine Richert, Oliver Dumele

**Affiliations:** ^1^ Institute of Chemistry Albert‐Ludwigs‐Universität Freiburg Alberstrasse 21 79104 Freiburg Germany; ^2^ Department of Chemistry Humboldt Universität zu Berlin Brook‐Taylor‐Strasse 2 12489 Berlin Germany; ^3^ EPR4Energy Joint Lab Department Spins in Energy Conversion and Quantum Information Science Helmholtz‐Zentrum Berlin für Materialien und Energie GmbH Albert‐Einstein‐Straße 16 12489 Berlin Germany; ^4^ Department of Chemistry and Biochemistry University of Cologne Greinstrasse 4 50939 Cologne Germany; ^5^ Present address: Institute of Physical and Theoretical Chemistry Goethe University Frankfurt Max‐von‐Laue‐Straße 7 60438 Frankfurt Germany

**Keywords:** Diradical, Electrochemistry, EPR spectroscopy, Photomagnet, Photoswitch

## Abstract

Controlling the spin state of a molecule using the spatiotemporal properties of visible light is of interest for spintronic devices in information technology or (bio)medical applications. We, herein, report an all‐organic visible light‐induced photochromic system than can switch from a diamagnetic (singlet) to a paramagnetic (triplet) state. This is realized by precisely tuning orbital symmetry and internal molecular strain in a [5]helicene scaffold substituted with an indanedione π‐acceptor. Irradiation with visible light at cryogenic temperatures gives a kinetically meta‐stable paramagnetic diradical state with a solvent‐dependent ground‐state multiplicity (triplet or singlet), which can be thermally switched back to its initial diamagnetic state.

## Graphical abstract

Visible light photochemical spin‐state switching is achieved through a tailored design. The photoswitching core is substituted with the π‐acceptor indanedione resulting in a small optical bandgap in combination with torsional twisting of the substituent. This twisting is essential for the stability of the diradical state as it isolates the spin density in the periphery preventing immediate radical–radical recombination.

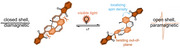

Precisely controlling the electronic spin state of molecular systems is an increasingly targeted scientific goal and has seen significant progress in recent years.^[^ ^1^, ^2^, ^3^, ^4^, ^5^, ^6^, ^7^, ^8^, ^9^ ^]^ The ability to reversibly direct between two electronic spin states, as well as their corresponding measurable chemical and physical properties, by applying an external influence is of importance in growing fields such as spintronics for (quantum) information technology^[^ ^2^, ^10^, ^11^, ^12^ ^]^ or (bio)medical^[^ ^1^, ^13^, ^14^, ^15^, ^16^ ^]^ purposes. In the last three decades, several contributions in the field have deepened our understanding of the (photo)chemical and ‐physical processes involved in spin‐state switching. Using electromagnetic waves as a spatiotemporally selective external stimulus has proven to be a useful method to generate a desired spin multiplicity.^[^ ^3^, ^4^, ^5^, ^6^, ^7^, ^9^, ^17^, ^18^, ^19^ ^]^ In principle, a molecular system is interchanged from a closed‐shell, diamagnetic singlet state to an open‐shell, paramagnetic triplet diradical(oid) state upon irradiation (Figure 1). An early example of light‐induced spin‐state switching of a purely organic material in the solid state was reported by Toda and Tanaka,^[^ ^17^ ^]^ followed by a small number of other reports achieving spin‐state switching in solution.^[^ ^6^, ^7^, ^18^ ^]^ All of these systems, however, only generated diradicals with a half‐life below two hours. More recently, Kubo and co‐workers as well as Feringa and co‐workers reported on photoconformational spin‐state switching achieving bistability of closed‐shell and open‐shell states.^[^ ^4^, ^5^ ^]^

**Figure 1:**
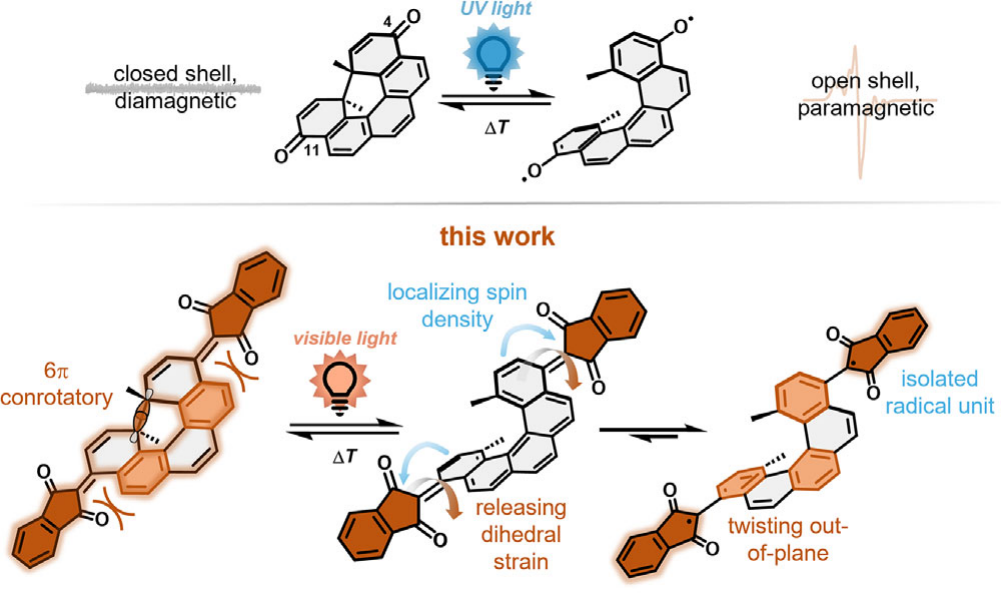
Schematic depiction of the photochemical spin‐state switching with UV light and visible light in this work.

Both molecular designs rely on the principle of strain release in highly strained anthraquinon derivatives. Our group has subsequently reported on helicene‐based photochemical magnetic switches with bistable spin state based on 6π electrocyclic ring‐opening and ‐closing reactions.^[^ ^3^ ^]^ These 4,11‐substituted helicenes achieve a chemically locked open‐shell diradical with triplet ground state by UV irradiation (Figure 1). Until now, all of the aforementioned systems rely on the use of UV light with high energy to achieve the spin‐state switching. Aiming for a visible light‐activated processes would not only reduce energy consumption but also enables a broader scope of applications. In particular, biological systems often rely on the use of low energy light and are not compatible with harsh UV light. However, the design of a low bandgap photochromic spin‐state switch based on pericyclic (photo)reactions is challenging due to the required orbital symmetry in both, the ring‐closed and ring‐open form. Installing a π‐accepting chromophore in the periphery of a photoswitching core leads to the delocalization of LUMO density away from the reactive center resulting in poor or no switching performance. On the other side, a diradical having a low optical bandgap will have nearly degenerate single occupied molecular orbitals (SOMOs). This causes spontaneous radical–radical recombination and a back reaction to the initial form—no bistability is achieved.

We herein report a concept of designing low bandgap photochemical spin‐state switches with bistable spin states by locating the diradical density at the exocyclic periphery, hindering the radical–radical recombination at the central reaction center. We realize this concept by a conformational twist of a dihedrally strained indanedione moiety after photochemical 6π retroelectrocyclization reaction at the helicene core. This locates the spin density of the radicals at the peripheral indandione. It leads to bistable spin states and switching operation with low fatigue over ten measured cycles using visible light. To realize absorbance in the visible range of the electromagnetic spectrum while ensuring radical stabilization and localization of the LUMO density, we envisioned substitution on the 4,11‐position of the [5]helicene scaffold with indanedione as an established acceptor chromophore unit.^[^ ^20^, ^21^ ^]^

Bis(indanedione) [5]helicene (±)‐**1**‐**C** was prepared using a Pd‐catalyzed C─H activation cross‐coupling method previously established by Wang and co‐workers.^[^ ^22^ ^]^ First, dibromo[5]helicene^[^ ^23^ ^]^ (±)‐**2** was reacted with indanedione under mild conditions using catalytic *t*Bu‐XPhos‐Pd G3^[^ ^24^ ^]^ to give open [5]helicene (±)‐**1**‐**O**‐**H_2_** in circa 75% yield along with small amounts of autoxidized closed form (±)‐**1**‐**C**. In contrast to the reported synthetic method, we found it crucial to perform the Pd‐catalyzed C─H coupling at temperatures as low as 40 °C (instead of 70 °C) to avoid dehalogenation of (±)‐**2**. The dihydro form (±)‐**1**‐**O**‐**H_2_** can be conveniently oxidized by air at 70 °C to give the closed form target (±)‐**1**‐**C** in an overall yield of 36% from (±)‐**2** (Scheme 1a). NMR spectroscopy studies of (±)‐**1**‐**O**‐**H_2_** suggest the presence of the keto tautomer (instead of the enol form) as indicated by a ^1^ *J* C─H coupling for the acidic indanedione proton in the ^1^H,^13^C‐HSQC NMR spectrum as well as two distinct C_Carbonyl_ signals in the ^13^C NMR spectrum originating from the two rotationally restricted carbonyl groups (Figure S29). Additionally, the exclusive presence of C═O stretch vibration resonances (and absence of any O─H stretch vibration bands) in the FT‐IR spectrum of (±)‐**1**‐**O**‐**H_2_** is clear evidence for the keto form (Figure S31).

**Scheme 1:**
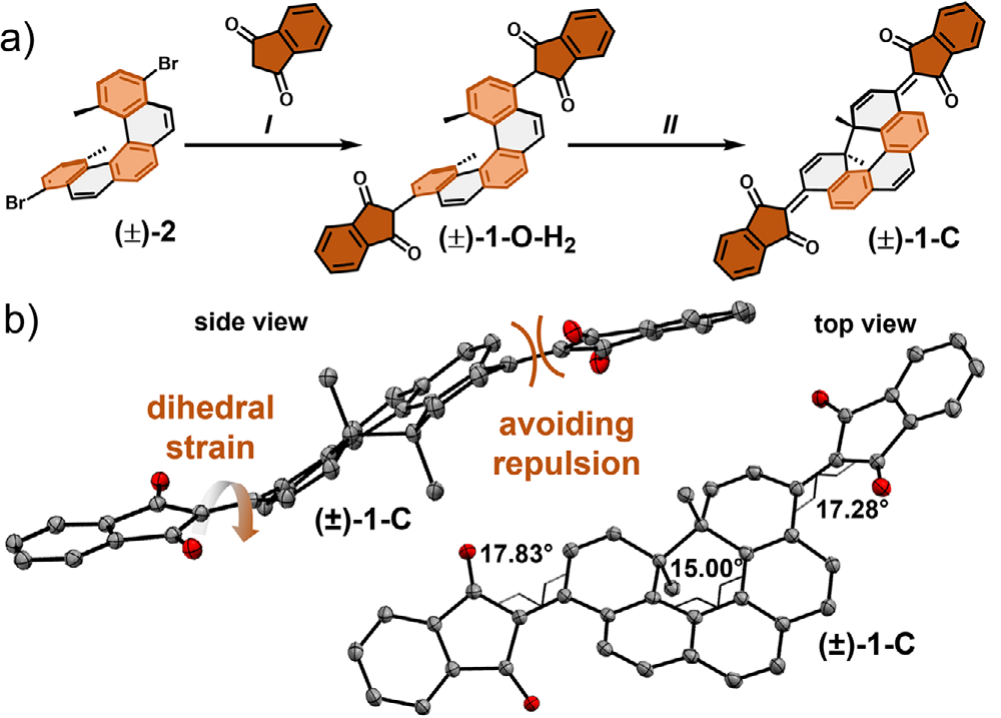
a) Synthesis of bis(indanedione) [5]helicene (±)‐1‐C. I) NaH, *t*Bu‐XPhos‐Pd G3, 1,4‐dioxane, 40 °C, 48 h. II) air, CHCl_3_/MeOH 1:1, 70 °C, 48 h, 36% over two steps; b) crystal structure of (±)‐1‐C in side and front view. Hydrogens and solvent molecules are omitted for clarity and ellipsoids are displayed at 50% probability level (bond lengths in Å).

The closed helical structure (±)‐**1**‐**C** was confirmed by ^1^H and two‐dimensional ^1^H,^13^C‐HMBC NMR spectroscopy with an unambiguous structural proof by single‐crystal X‐ray diffraction (SCXRD). The indanedione substituent is significantly twisted out of the helical plane to reduce repulsion, resulting in a C─C═C─C angle of 17.28° and 17.83° (Scheme 1b). In contrast, the previously reported analogous bis(dicyanomethylidene) shows a nearly coplanar substitution (average C─C═C─C angle of 1.2° and 3.1°).^[^ ^3^ ^]^

The UV–vis spectrum of (±)‐**1**‐**C** confirms the envisioned absorbance in the visible light region with *λ*_max_  = 480 nm and a derived bandgap of Δ*E*_opt_ = 2.39 eV. To examine the spin‐state switching properties of (±)‐**1**‐**C** with visible light, (±)‐**1**‐**C** was irradiated close to its longest wavelength absorption with 450 nm LED light, monitored by UV–vis spectroscopy at 77 K, in a glassy 2‐methyl tetrahydrofuran (2‐MeTHF) matrix (Figure 2a). Irradiation to the photostationary state (PSS) over 50 min led to the formation of broad long‐wavelength absorbance bands reaching to 650 nm, typically associated with open‐shell diradical systems.^[^ ^3^, ^25^, ^26^, ^27^, ^28^, ^29^, ^30^ ^]^ Irradiation at room temperature for 50 min did not lead to the formation of long‐wavelength bands (Figure S3). Even though the newly emerged bands show a significant redshift, a fully delocalized diradical bearing a chromophore would be expected to exhibit bands far further redshifted then observed here. This confirms our design principle, in which the indanedione readily twists out of plane upon ring opening, releasing the dihedral strain and localizing the spin density away from the helicene core (Figure 2d). This interpretation is further supported by the computed spin density map of (±)‐**1**‐**C** showing strong localization on the indanedione (Figure S10). An isosbestic point at 498 nm was retained for the 50 min duration of irradiation suggesting a clean conversion of (±)‐**1**‐**C** into its ring‐open form (±)‐**1**‐**O**. Attempts to trigger photochemical back reaction at various wavelengths remained unsuccessful, but warming up the glassy matrix above 95 K leads to the reverse ring‐closing of (±)‐**1**‐**O**, and recovers the initial UV–vis spectrum of (±)‐**1**‐**C** after completely thawing the glassy matrix (Figure 2b). This cycle can be repeated several times with a constant amount of (±)‐**1**‐**O** generated each time, suggesting good fatigue resistance of the photochromic system (Figure 2b). In contrast to systems relying on spin‐state changes in photoexcited states, the bistability below 95 K allows to address the two different spin states selectively for a chosen time span, orders of magnitude larger than for photoexcited triplets.

**Figure 2:**
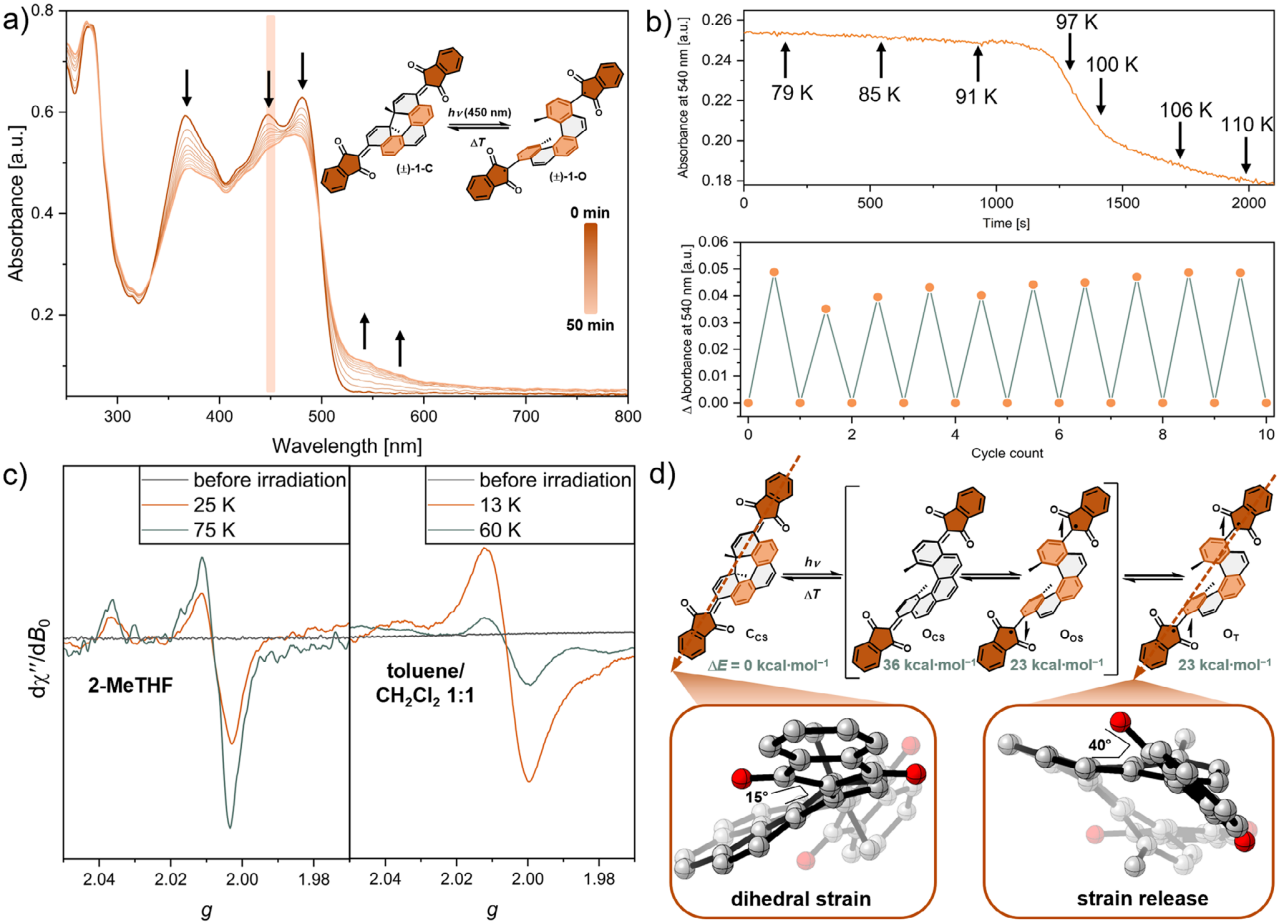
a) UV–vis spectrum of (±)‐1‐C during irradiation with 450 nm visible light for 50 min at 77 K in a glassy matrix of 2‐MeTHF (c = 1.4·10^−5^ M); b) thermal back reaction of (±)‐1‐O followed at 540 nm and heating with 1 K·min^−1^ (top) reversible switching cycles (50 min irradiation @450 nm, warming to 200 K, cooling back to 77 K) monitored by the difference in absorbance at 540 nm (bottom); c) VT‐EPR spectrum of (±)‐1‐O in 2‐MeTHF (left) and toluene/CH_2_Cl_2_ 1:1 (right, *c* ∼ 10^−4^ M); d) Proposed reaction mechanism upon irradiation of (±)‐1‐C with relative computed energies and geometries of (±)‐1‐C_CS_ and (±)‐1‐O_T_ (level of theory: DFT‐UB3LYP/def2‐SVPP). The EPR spectra in panel C are smoothed, original data, and experimental conditions can be found in the Supporting Information.

Motivated by these initial spectroscopic findings, we conducted electron paramagnetic resonance (EPR) spectroscopy to i) verify the formation of a paramagnetic diradical upon irradiation and ii) probe the ground‐state spin multiplicity of (±)‐**1**‐**O**. At 77 K, nonirradiated (±)‐**1**‐**C** was found to be EPR‐silent, as expected for the diamagnetic closed‐shell form (Figure 2c). Irradiation with 450 nm LED light for 60 min resulted in an EPR signal at *g* ≈ 2.0, typical for organic radicals (Figure 2c).^[^ ^31^, ^32^ ^]^ Variable‐temperature (VT‐)EPR spectroscopy of a previously irradiated sample of (±)‐**1** in toluene/CH_2_Cl_2_ 1:1 in the range of 13–80 K indicated a triplet ground state as the EPR intensity decreases continuously with increasing temperature (Figures S6 and S8). Interestingly, the spin system is changed to a singlet ground state in a matrix of 2‐MeTHF (Figures S7 and S8). Upon rising the temperature from 25 K, the intensity increases to a maximum at 75 K as the excited triplet state becomes increasingly populated, before the decrease sets above 75 K (Figures S7 and S8). It has been observed that polar solvents with high dielectric constants increase the stability of the singlet state, explaining the observed solvent dependency of the ground‐state spin multiplicity of (±)‐**1**‐**O**.^[^ ^33^, ^34^ ^]^ The fact that the EPR signals do not exhibit a visible splitting due to spin–spin exchange, respectively a typical triplet state shape, indicates that the exchange interaction constant is small, i.e., within the line width. The triplet spin state of paramagnetic (±)‐**1**‐**O** was verified by nutation experiments revealing a nutation frequency of $\sqrt{2}$ *ω*_0_ characteristic for triplet state transitions, where ω_0_ is the reference frequency of a species with doublet multiplicity (Figure S9).

The spin‐state switching behavior, from a diamagnetic closed‐shell (±)‐**1**‐**C** to a paramagnetic open‐shell triplet (±)‐**1**‐**O**, was rationalized using DFT calculations. In its initial state, (±)‐**1**‐**C** is expected to be in its closed‐shell electronic configuration due to sufficient stabilization through the two Clar's sextets.^[^ ^35^ ^]^ After irradiation and Woodward–Hoffmann (WH)‐allowed 6π retroelectrocyclization,^[^ ^36^ ^]^ a ring‐open closed‐shell (**O_CS_**) configuration deprived of all Clar's sextets can be formed. Additionally, two ring‐open radical forms are possible: an open‐shell singlet (**O_OS_**) or triplet (**O_T_**) configuration with three Clar's sextets each (Figure 2d).^[^ ^35^, ^36^, ^37^, ^38^, ^39^ ^]^ Intuitively, a ring‐open closed‐shell configuration is expected to be significantly destabilized (*E*_rel_(**O_CS_**)  = 36 kcal·mol^−1^) compared to the ring‐closed form (±)‐**1**‐**C**. The two open‐shell configurations are predicted to have significantly lower relative energies with *E*_rel_(**O_OS_**) ≈ *E*_rel_(**O_T_**) = 23 kcal·mol^−1^, which is in agreement with the observed small—but solvent‐dependent—singlet–triplet energy gap. The inaccessibility of the ring‐open closed‐shell configuration (**O_CS_**) also prevents a WH‐allowed photochemical reaction back to (±)‐**1**‐**C**.

We were also interested in the electrochemical switching behavior between (±)‐**1**‐**C** and an open bis‐anion (±)‐**1**‐**O^2−^**. The cyclic voltammogram (CV) of (±)‐**1**‐**C** shows an irreversible cathodic peak (*E*_p_^c^ = –1.118 V versus Fc/Fc^+^, scan rate 0.05–1.00 V·s^−1^, Figure 3a) corresponding to a two‐electron reduction forming the ring‐open bis‐anion (±)‐**1**‐**O^2−^** via an ECE mechanism.^[^ ^40^ ^]^ The ECE mechanism is characterized by the initial abstraction of an electron to form the radical anion (±)‐**1**‐**C^– ·^**, which rapidly undergoes ring‐opening to (±)‐**1**‐**O^– ·^** as the chemical reaction step, followed by the abstraction of a second electron resulting in the final bis‐anion (±)‐**1**‐**O^2−^**. The ring‐open [5]helicene bis‐anion can be reoxidized to the ring‐closed (±)‐**1**‐**C** at scan rates above 0.5 V·s^−1^ with an anodic potential of *E*_p_^a^ = –0.151 V versus Fc/Fc^+^(for a full discussion, see Supporting Information, Section S6). The electrochemical reduction of (±)‐**1**‐**C** was followed by in situ UV–vis spectroscopy showing a decrease of the bands at 460 and 365 nm and an increase at 300 and 330 nm, respectively, in the potential range of −0.8 to −1.7 V versus Fc/Fc^+^ (Figure 3a, top). During reoxidation of (±)‐**1**‐**O^2−^** to (±)‐**1**‐**C** in the potential range of −0.24 to 0.4 V versus Fc/Fc^+^ the spectral change is reversed (Figure 3a, bottom). Due to diffusion limitations, the reoxidation is not quantitative.

**Figure 3:**
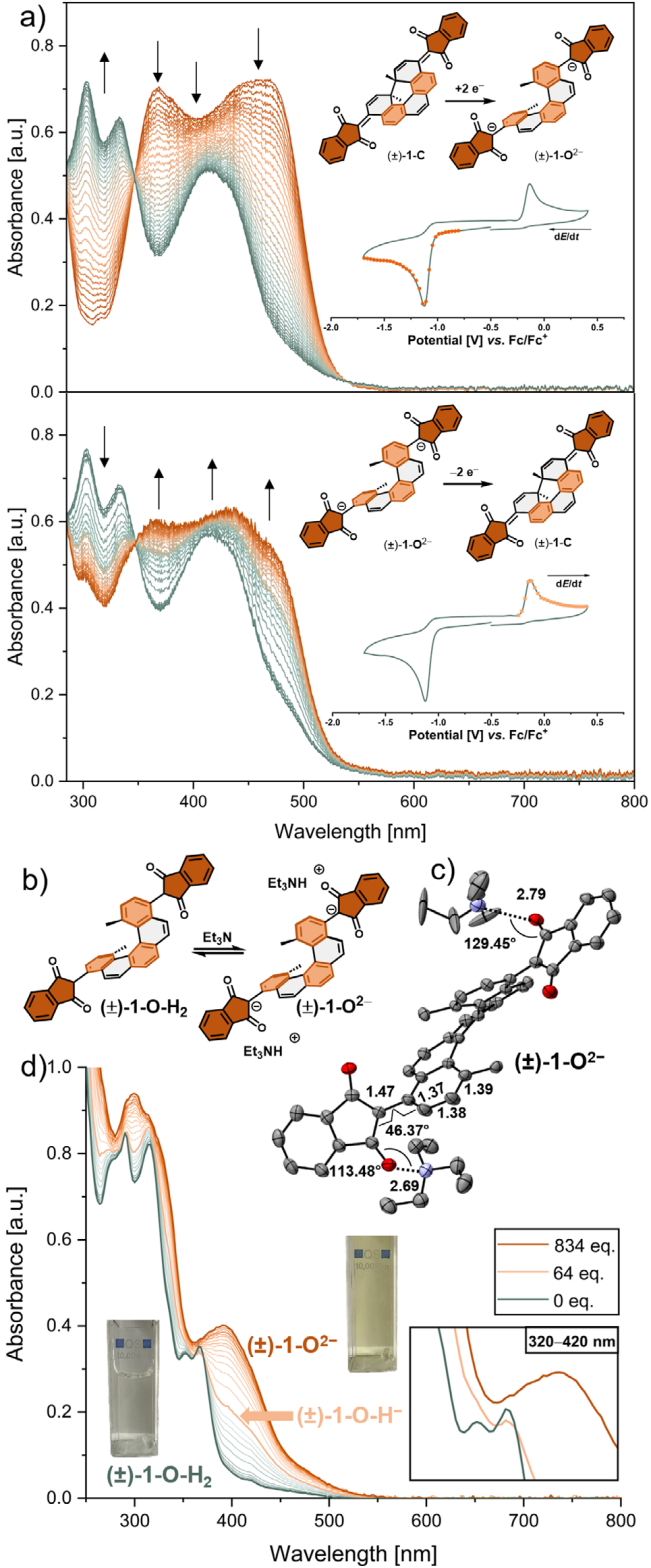
a) Spectroelectrochemical reduction (top) and reoxidation (bottom) of (±)‐1‐C in dimethylformamid (*c* = 0.3 mM). Inset shows the CV spectra with dots and squares indicating the potentials at which spectra were recorded; b) acid base equilibrium between (±)‐1‐O‐H_2_ and (±)‐1‐O^2−^; c) crystal structure of (±)‐1‐O^2−^. Hydrogens and solvent molecules are omitted for clarity and ellipsoids are displayed at 50% probability level (bond lengths and distances in Å); d) UV–vis titration of (±)‐1‐O‐H_2_ with Et_3_N in THF (*c *= 2.3·10^˗5^ M in THF at 298 K). Picture insets show the analyte solution before and after addition of excess Et_3_N.

To obtain structural insights of the open helicene species, the bis‐anionic species of bis(indanedione) [5]helicene, (±)‐**1**‐**O^2–^**, was generated by deprotonation of (±)‐**1**‐**O**‐**H_2_** in solution monitored by UV–vis spectroscopy titration with Et_3_N (Figure 3b,d). The addition of Et_3_N to a solution of (±)‐**1**‐**O**‐**H_2_** in THF quickly leads to the formation of the mono anion (±)‐**1**‐**O**‐**H^–^**, followed by further deprotonation to (±)‐**1**‐**O^2–^** (Figure 3d). To our satisfaction, single crystals of (±)‐**1**‐**O^2–^** with its two triethylammonium counter ions were obtained by slow evaporation of a solution of (±)‐**1**‐**O**‐**H_2_** with a few drops Et_3_N in CH_2_Cl_2_/toluene under a N_2_ atmosphere (Figure 3c). Short heavy‐atom distances of 2.79 and 2.69 Å and highly directional N─O─C angles of 129.45° and 113.48° indicate strong hydrogen bonding.^[^ ^41^ ^]^ In contrast to closed form [5]helicene (±)‐**1**‐**C**, no significant bond length alternation of the terminal benzene ring is observed, hence a fully delocalized π‐system is present. The indanedione substituent is significantly twisted out of plane with a dihedral angle of 46.37°, which further confirms our initial molecular design based on strain release.

In conclusion, we have presented a concept for photochemical spin‐state switching using visible light (450 nm). The small bandgap was realized by installing indanedione as a π‐acceptor while ensuring a sufficiently high electron density on the photoswitching [5]helicene core. The system can be reversibly converted from a closed‐shell singlet state to an open‐shell triplet diradical state by irradiation with visible light. The initial state is thermally recovered. Spin bistability could be achieved by releasing dihedral strain and localizing the spin‐density onto the indanedione unit upon ring‐opening. Additionally, we were able to generate a rare stable bis‐anion by deprotonation or electrochemically.^[^ ^42^, ^43^, ^44^ ^]^ The ability to selectively address a metastable spin state over an extended period of time sets photochemical spin‐state switches apart from systems that rely on a transient change in spin state, such as photoexcited acene triplets. We envision these photogenerated diradical states with persistent life times become viable candidates as molecular qubits, in quantum sensing, or (bio)medical contexts.

## Supporting Information

The authors have cited additional references within the Supporting Information.^[^ ^3^, ^23^, ^45^, ^46^, ^47^, ^48^, ^49^, ^50^, ^51^, ^52^, ^53^, ^54^, ^55^, ^56^, ^57^, ^58^, ^59^, ^60^ ^]^

## Conflict of Interests

The authors declare no conflict of interest.

## Supporting information



Supporting Information

Supporting Information

## Data Availability

The data that support the findings of this study are available in the Supporting Information of this article.
