# Supplementary material for: Photochemical Spin‐State Switching of an All‐Organic Molecular System with Visible Light
Source: Angew Chem Int Ed Engl. 2025 Sep 26;64(46):e202515144. doi: 10.1002/anie.202515144 (PMC12603987; doi:10.1002/anie.202515144)
Supplement: Supplementary file 1 — Supporting Information [file ANIE-64-e202515144-s002.pdf]

Supporting Information

for

Photochemical Spin-State Switching of an all-organic Molecular System with Visible Light

Joël Schlecht,[a,d] Thomas Lohmiller,[b,c] Philipp Thielert,[a] Clara Douglas,[a,d] Malte Gather,[d] Sabine Richert,[a,e] and Oliver Dumele[a,d]*

[a] J. Schlecht, C. Douglas, P. Thielert, Prof. Dr. S. Richert, Prof. Dr. O. Dumele
Institute of Chemistry
Albert-Ludwigs-Universität Freiburg
Alberstrasse 21, 79104 Freiburg, Germany

[b] Dr. T. Lohmiller
Department of Chemistry
Humboldt Universität zu Berlin
Brook-Taylor-Strasse 2, 12489 Berlin, Germany

[c] Dr. T. Lohmiller
EPR4Energy Joint Lab, Department Spins in Energy Conversion and Quantum Information Science
Helmholtz-Zentrum Berlin für Materialien und Energie GmbH
Albert-Einstein-Straße 16, 12489 Berlin, Germany

[d] J. Schlecht, C. Douglas, Prof. Dr. M. Gather, Prof. Dr. O. Dumele
Department of Chemistry and Biochemistry
University of Cologne
Greinstrasse 4, 50939 Cologne, Germany
Email: odumele@uni-koeln.de
www.dumelelab.com

[e] Present address: Institute of Physical and Theoretical Chemistry
Goethe University Frankfurt
Max-von-Laue-Straße 7, 60438 Frankfurt, Germany

Table of Content

S1. Materials and Instrumentation 2

S2. Synthetic procedures 6

S3. Additional UV-vis Spectroscopy 10

S4. (VT)-EPR Spectroscopy 13

S5. Computational Details 16

S6. (Spectro)electrochemistry 17

S7. Crystallographic Data 21

S8. Selected NMR Spectra 25

S9. Selected ATR-FT-IR Spectra 33

S10. Cartesian Coordinates 34

S11. References 42

S1. Materials and Instrumentation

Reagents (Acros, AlfaAesar, BLDpharm, Sigma-Aldrich, and TCI) were purchased as reagent grade and used without further purification, unless otherwise specified.

Solvents for synthesis were dried using a Pure Solv Micro Solvent Purification System from Innovative Technology and stored over molecular sieves 3–4 Å.

Glassware. All non-aqueous reactions were performed in oven-dried glassware and under an N_2 atmosphere.

Automated medium pressure column chromatography (MPLC) was performed on a Teledyne ISCO CombiFlashRf 300 system with 200 mL min^{-1} max flow, 200 psi, equipped with integrated ELSD and 200–800 nm UV-vis variable wavelength detector.

High-performance liquid chromatography (HPLC) was run on a Waters 600 HPLC System equipped with a waters 600 solvent pump, a Waters 600 controller, and a waters 996 photodiode array detector.

UPLC–HR-ESI-MS was performed using a Waters UPLC ACQUITY H-Class PLUS with a Waters Alliance System (Waters Separations Module 2695, Waters Diode Array Detector 996) coupled to a Xevo G3 QTof mass analyzer with ESI or API ion source.

Thin layer chromatography (TLC) was conducted on aluminum sheets coated with SiO_2-60 F_{254} obtained from Merck; visualization with a UV lamp (254 or 366 nm).

Nuclear magnetic resonance (NMR) spectra were recorded using a Bruker Avance II 300 (300 MHz for ^1H and 75 MHz for ^{13}C) and a Bruker Avance II 500 (500 MHz for ^1H and 126 MHz for ^{13}C) at 25 °C and are reported as follows: chemical shift (δ) in ppm (multiplicity, coupling constant *J* in Hz, number of protons; assignment). The residual deuterated solvent was used as the internal reference ($CDCl_3$: δ_H = 7.26 ppm); ($CDCl_3$: δ_C = 77.16 ppm); The resonance multiplicity is described as s (singlet), d (doublet), t (triplet), q (quartet), m (multiplet), and br. (broad).

Infrared (IR) spectra were recorded on a Perkin-Elmer Spectrum Two FT-IR equipped with a diamond ATR attachment and are basegraph-corrected. The spectra were measured between 4000 and 500 cm^{-1} with 16 scans. Absorbance bands are reported in

wavenumbers (cm^{-1}) and their relative intensities described as s (strong), m (medium), or w (weak).

Single-crystal X-ray data were measured with a BRUKER D8 VENTURE area detector with Mo-Kα radiation (λ = 0.71073 Å). Multi-scan Absorbance corrections implemented in SADABS[1] were applied to the data. The structures were solved by intrinsic phasing method (SHELXT-2013)[2] and refined by full matrix least square procedures based on F2 with all measured reflections (SHELXL-2014)[3] in the graphical user interface (SHELXLe)[4] with anisotropic temperature factors for all non-hydrogen atoms. All hydrogen atoms were added geometrically and refined by using a riding model.

Ultraviolet–Visible (UV-vis) absorbance spectroscopy was performed on Agilent Cary 60 instruments connected to a cryostat from Unisoku Scientific Instruments (temperature accuracy ±0.1 K) in 10 × 10 mm quartz cuvettes with 3 mL volume. Weighing of small quantities was performed on a Sartorius ME5 analytical microbalance.

Variable temperature Ultraviolet–Visible (VT-UV-vis) absorbance spectroscopy was performed on Agilent Cary 60 instruments equipped with a OptistatDN from Oxford Instruments connected with a MercuryiTC temperature controller from Oxford Instruments in a 221.001-QS quartz cuvette from neoLab with 3 mL volume.

Steady state fluorescence was measured with a Varian Cary Eclipse Fluorescence Spectrometer equipped with a 80 Hz xenon flash lamp and Peltier thermostat module in 10 × 10 mm quartz cuvettes with 3 mL volume.

Continuous wave electron paramagnetic resonance (EPR) spectroscopy at X-band frequencies was performed on a Bruker EMXplus instrument equipped with a cryogen-free closed cycle helium recirculating cooling system from ColdEdge for (±)-**1-O** in toluene/CH_2Cl_2 1:1, and on a Bruker ELEXSYS E580 spectrometer with a Bruker ER 4118X-MD5 resonator and an Oxford Instruments ER 4118CF helium flow cryostat for (±)-**1-O** in 2-MeTHF. Samples were measured at several temperatures from 13 to 80 K (toluene/CH_2Cl_2) and 25 to 90 K (2-MeTHF) as glassy frozen solutions (powder spectra). At each temperature point, the microwave power was chosen such as to exclude saturation of the EPR transition upon testing the saturation behaviour using a series of powers.

Pulse EPR measurements at Q-band frequencies were performed on a Bruker ELEXSYS E580 spectrometer equipped with a Bruker EN45107D2 resonator and an Oxford Instruments nitrogen gas-flow cryostat (CF 935). The measurements were performed in 2-MeTHF as a glassy frozen solution at a constant temperature of 80 K. The stable radical species was generated directly at 80 K inside the spectrometer before the measurements, by irradiation of the sample through the top of the sample holder with depolarised light at 450 nm for 5 hours using an optical fiber with a diameter of 0.8 mm and a pulsed laser with an excitation energy of ~0.5 mJ at a repetition rate of 50 Hz (pulse duration ~5 ns). The measurements were afterwards performed in the dark. The echo-detected field-swept EPR spectrum was recorded using a standard Hahn echo sequence ($\pi/2 - \tau - \pi - \tau -$ echo) with τ = 200 ns and a π-pulse length of 32 ns. A two-step phase cycle was applied. The spectrum was frequency-corrected to 34.0 GHz and field-corrected using a carbon fiber standard with g = 2.002644.[5] Nutation measurements were performed using the sequence $\xi - \tau - \pi -$ echo where the flip angle ξ was gradually increased by increasing the corresponding microwave pulse length in steps of 2 ns, starting at 16 ns ($\pi/2$). The integrated echo intensity was recorded as a function of this ξ-pulse length at specific magnetic field positions within the region of the spectrum. The data were background corrected using a first-order polynomial function. After dead-time reconstruction, windowing using a Hamming window, and zero filling to 1024 data points, the cross-term averaged Fourier transform was calculated. The resulting frequency spectra were normalized by division of the frequency axis by the reference frequency (ω_0) obtained for the glass artefact signal (doublet multiplicity).

Cyclic voltammetry (CV) was performed using a PG310 USB (HEKA Elektronik) potentiostat interfaced to a PC with PotMaster v2x43 (HEKA Elektronik) software for data evaluation. A three-electrode configuration contained in a nondivided cell consisting of a glassy carbon disc (d = 1.0 mm) as the working electrode, a platinum plate as the counter electrode, and a saturated calomel electrode (SCE) with an agar–agar plug in a Luggin capillary with a diaphragm as the reference electrode was used. Measurements were carried out in DMF containing 0.1 M Bu_4NPF_6 using various scan rates. The data is given in reference to the ferrocene redox couple (Fc/Fc^+), which was used as an external standard.

UV-vis acid–base titration was performed by preparing a parent solution of (±)-**1-OH$_2$** in THF. Of this solution, 2 mL were transferred into a 10 × 10 mm quartz cuvette while another 2 mL were treated with a 1000-fold excess of triethylamine. The triethylamine solution was then used to titrate the analyte solution.

Spectroelectrochemistry was performed in a SEC-C Thin Layer Quartz Glass Spectroelectrochemical cell with a 1.0 mm optical path length, with platinum mesh electrode as a working electrode, counter electrode: platinum wire, reference electrode: non aqueous reference electrode Ag/Ag+ (0.01 M AgNO3 in 0.1 M Bu4NPF6 acetonitrile), ALS Co., Ltd (Tokyo, Japan) spectrometer: Metrohm ASM80120 with ALS80112 light source Potentiostat: PGSTAT 204, Deutsche Metrohm GmbH & Co. KG (Filderstadt, Germany) and software: NOVA 2.1. Scan rate for all measurements dE/dt = 10 mV s^{-1}.

Nomenclature for the open-form [5]helicenes was performed according to the common numbering, where the two fjord-positions are numbered as 1 and 14. According to IUPAC rules, these positions are numbered as 10 and 11, but were not adopted for the sake of simplicity. The closed-form [5]helicenes were numbered in the same way instead of using the IUPAC rules for perylenes.

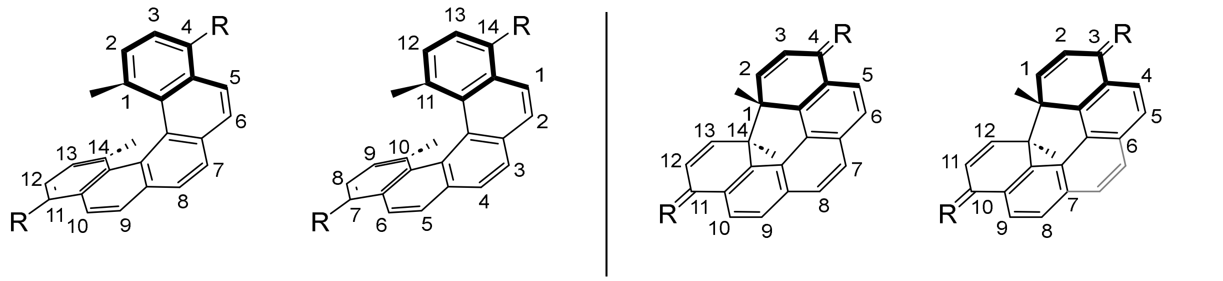

Common numbering IUPAC numbering Chosen numbering IUPAC numbering

S2. Synthetic procedures

1,4-Bis(triphenylmethylenphosphonium)benzene dibromide (3)[6]

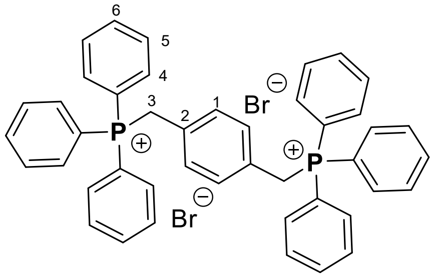

Based on a procedure reported in the literature,[6] 1,4-bis(bromomethyl)benzene (50.00 g, 189.40 mmol) and triphenylphosphine (105.00 g, 397.80 mmol) were dissolved in dry DMF (500 mL) and heated to 150 °C for 1 h. The suspension was cooled to 23 °C, filtered over a Büchner funnel and washed with an excess of diethylether. The product was dried under reduced pressure to yield **3** (141 g, 95%, Lit.:[6] 97%) as a colorless powdery solid.

The spectral data are in line with the literature.[6] **^{1}H NMR** (500 MHz, $CDCl_3$, 25 °C, assignments based on ^{1}H,^{1}H-COSY, ^{1}H,^{13}C-HSQC and ^{1}H,^{13}C-HMBC NMR spectra): δ = 7.75–7.62 (m, 30H, H–C(4,5,6)), 6.93 (s, 4H, H–C(1)), 5.37 ppm (d, J = 13.3 Hz, 4H, $H_2C(3)$); **^{13}C NMR** (126 MHz, $CDCl_3$, 25 °C): δ = 135.3, 134.5, 132.1, 130.4, 117.9, 117.2, 30.3 ppm.

1,4-Bis(2-bromo-5-methylstyryl)benzene (4)[7]

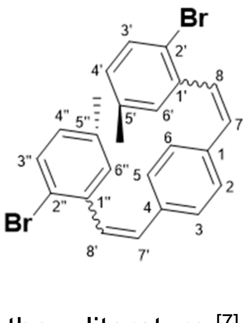

Adapted with changes from the literature,[7] a suspension of 2-bromo-5-methylbenzaldehyde (20.00 g, 100.48 mmol, 2 eq), **3** (40.00 g, 50.73 mmol, 1.01 eq), and 18-crown-6 (9.30 g, 35.167 mmol, 0.7 eq) in CH_2Cl_2 (700 mL) was degassed for 15 min using a stream of argon. Freshly ground KOH (12.97 g, 231.10 mmol, 4.6 eq) was added and the resulting mixture was stirred at 20 °C for 15 h. The solvent was removed under

reduced pressure and the residue was suspended with cyclohexane/EtOAc (95:5, 1000 mL). The suspension was filtered over a silica plug and the filtrate was concentrated under reduced pressure to afford **4** (23.06 g, 98%, Lit:[7] 90%) as a yellow oil.

***R*f** = 0.39 (SiO_2; cyclohexane); **^{1}H NMR** (500 MHz, CD_2Cl_2, 25 °C): δ = 7.59–7.38 (m) 7.18–6.92 (m), 6.68–6.54 (m), 2.36 (s, (*trans*,*trans*) isomer), 2.34 (s, (*cis*,*trans*) isomer), 2.17 (s, (*cis*,*trans*) isomer), 2.14 ppm (s, (*cis*,*cis*) isomer) (the sum of the integrals in the range of 7.59–6.54 ppm correspond to the number of aromatic (10) and olefinic (4) protons, as does the sum of the integrals in the range of 2.36–2.14 ppm corresponds to the number of protons of the methyl groups (6); **^{13}C NMR** (126 MHz, CD_2Cl_2, 25 °C): δ = 138.4, 138.2 (d), 137.9, 137.7, 137.1, 136.8, 136.7, 136.1, 133.3, 133.0, 132.9, 131.8 (d), 131.3 (d), 131.2, 130.5, 130.3 (d), 130.1, 129.9, 129.3, 127.8 (d), 127.8, 127.1, 121.4, 121.3, 120.8, 120.7, 21.3 (d), 21.1 ppm (d), (the amount of signals results from the different isomers).

(±)-4,11-Dibromo-1,14-dimethyldibenzo[*c*,*g*]phenanthrene ((±)-2)[7]

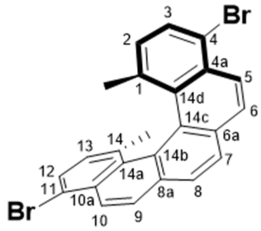

In three separate 2 L-three-neck round-bottom flasks each, a solution of **6** (1.500 g, 3.20 mmol, 1 eq) and *N*-iodosuccinimide (1.586 g, 7.05 mmol, 2.2 eq) in THF (36.4 mL, 448.50 mmol, 140 eq) was diluted with cyclohexane (2000 mL) and degassed for 15 min using a stream of nitrogen. The reaction was stirred and irradiated for 16 h at 30 °C using three LED panels for each reaction flask (20 cm x 16 cm, 45 single LED's, 50 W, 395 nm peak emission). The combined reaction mixture of all three flasks was concentrated under reduced pressure. The red sticky residue was redissolved in CH_2Cl_2 (80 mL), diluted with cyclohexane (80 mL), and eluted through a solid $Na_2S_2O_3$-layered round silica plug (8 cm x 4 cm SiO_2, 8 cm x 0.5 cm $Na_2S_2O_3$). Residual product was eluted using cyclohexane/CH_2Cl_2 9:1 (250 mL). The filtrate was concentrated under reduced pressure and redissolved in CH_2Cl_2 (80 mL), filtered over cotton into a crystallization dish, and

diluted with toluene (8 mL). Slow evaporation over 48 h afforded (±)-**2** (504 mg, 34%, Lit.:[7] 73%) as a yellow crystalline solid.

***R*f** = 0.55 (SiO_2; cyclohexane); the spectral data are in agreement with the literature.[7] **^{1}H NMR** (500 MHz, $CDCl_3$, 25 °C, assignments based on ^{1}H,^{1}H-COSY, ^{1}H,^{13}C-HSQC, and ^{1}H,^{13}C-HMBC NMR spectra): δ = 8.40 (d, *J* = 8.7 Hz, 2H, H–C(5,10)), 8.08 (s, 2H, H–C(7,8)), 8.00 (d, *J* = 8.7 Hz, 2H, H–C(6,9)), 7.78 (d, *J* = 7.8 Hz, 2H, H–C(3,12)), 6.95 (d, *J* = 7.8 Hz, 2H, H–C(2,13)), 0.89 ppm (s, 6H, H_3C–C(1,14)); **^{13}C NMR** (126 MHz, $CDCl_3$, 25 °C, assignments based on ^{1}H,^{1}H-COSY, ^{1}H,^{13}C-HSQC, and ^{1}H,^{13}C-HMBC NMR spectra): δ = 134.7 (C(1,14)), 133.9 (C(14a,14d), 131.5 (C(6a,8a)), 131.4 (C(4a,10a)), 130.2 (C(3,12)), 129.3 (C(2,13)), 127.1 (C(6,9)), 127.0 (C(7,8)), 126.6 (C(5,10)), 126.2 (C(14b,14c)), 119.9 (C(4,11)), 22.4 ppm (CH_3).

(±)-2,2'-(7a,7b-Dimethyl-7a,7b-dihydrobenzo[*ghi*]perylene-5,10-diylidene)bis(1H-indene-1,3(2H)-dione) ((±)-1-C)

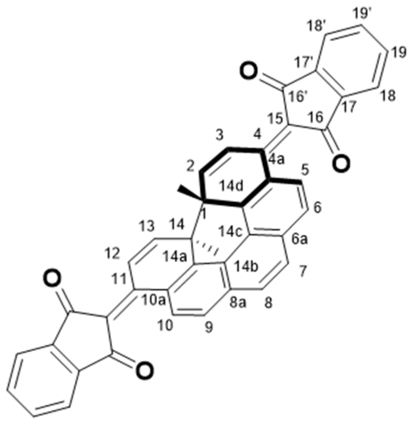

A solution of dibromo [5]helicene (±)-**5** (50 mg, 0.11 mmol), sodium hydride (30 mg, 0.75 mmol, 60% dispersion in mineral oil), and 1H-indene-1,3(2H)-dione (81 mg, 0.54 mmol) in dry 1,4-dioxane (2.2 mL) in an oven-dried pressure tube was degassed for 10 min using a stream of N_2. The mixture was treated with [(2-di-*tert*-butylphosphino-2',4',6'-triisopropyl-1,1'-biphenyl)-2-(2'-amino-1,1'-biphenyl)][palladium(II)-methansulfonat (*t*BuXPhos Pd G3) (6.3 mg, 7.5 μmol), the tube was sealed, and placed in an oil bath at 40 °C for 48 h. After cooling to 23 °C, the reaction mixture was diluted with 0.05 M aq. HCl (50 mL), extracted with CH_2Cl_2 (3 x 30 mL), washed with brine (50 mL), and dried over

anh. $MgSO_4$. After evaporating to dryness, the crude product was purified using manual column chromatography (~50 g SiO_2, cyclohexane/ethyl acetate 1:1) to yield an orange solid which still contains circa 10% of 1H-indene-1,3(2H)-dione. The pure form can be obtained by repeating the last purification step. For the following oxidation, the compound can be used as obtained. The yellow solid was dissolved in $CHCl_3$/methanol 1:1 (100 mL) and heated to reflux for 48 h under air. After concentrating the mixture to dryness, the crude product was purified by preparative MPLC (40 g SiO_2, prepacked from Teledyne ISCO, cyclohexane/ethyl acetate 7:3) to yield (±)-**1-C** (23 mg, 36% over two steps) as a bright orange solid.

R_f = 0.37 (cyclohexane/ethyl acetate 7:3); **^{1}H NMR** (500 MHz, $CDCl_3$, 25 °C, assignments based on ^{1}H,^{1}H-COSY, ^{1}H,^{13}C-HSQC, and ^{1}H,^{13}C-HMBC NMR spectra): δ = 8.95 (d, *J* = 10.2 Hz, 2H, H–C(3,12)), 8.31 (d, *J* = 8.4 Hz, 2H, H–C(5,10)), 8.01–7.98 (m, 2H, H–C(18)), 7.98 (s, 2H, H–C(7,8)), 7.97–7.95 (m, 2H, H–C(18')), 7.91 (d, *J* = 8.5 Hz, 2H, H–C(6,9)), 7.81–7.74 (d, 4H, H–C(19,19')), 7.51 (d, *J* = 10.4 Hz, 2H, H–C(2,13)), 1.16 ppm (s, 6H, H_3C–C(1,14)); **^{13}C NMR** (126 MHz, $CDCl_3$, 25 °C): δ = 192.4 (C=O(16)), 189.5 (C=O(16')), 151.9 (C(15)), 145.0 (C(2,13)), 143.3 (C(14a,14d)), 141.3 (C(17,17')), 135.0 and 134.9 (C(19,19')), 133.3 (C(6a,8a)), 131.4 (C(5,10)), 129.5 (C(7,8)), 127.5 (C(3,12)), 127.2 (C(4a,10a)), 124.7 (C(6,9)), 124.4 (C(14b,14c)), 123.1 (C(18')), 122.8 (C18)), 122.6 (C(15)), 45.8 (C(1,14)), 27.1 ppm (CH_3); **FTIR (ATR)**: $\tilde{\nu}_{max}$ = 3006 (m), 2989 (m), 1662 (m), 1626 (w), 1591 (w), 1509 (m), 1276 (s), 1262 (s), 1219 (w), 1129 (w), 1105 (w), 1064 (w), 767 (s), 741 cm^{-1} (s); **HR-ESI-TOF-MS**: *m*/*z*: 593.1749 ([*M* + H]$^+$ calcd. for $C_{42}H_{25}O_4^+$: 593.1747). Unambiguous structural proof is provided by single-crystal X-ray diffraction.

S3. Additional UV-vis Spectroscopy

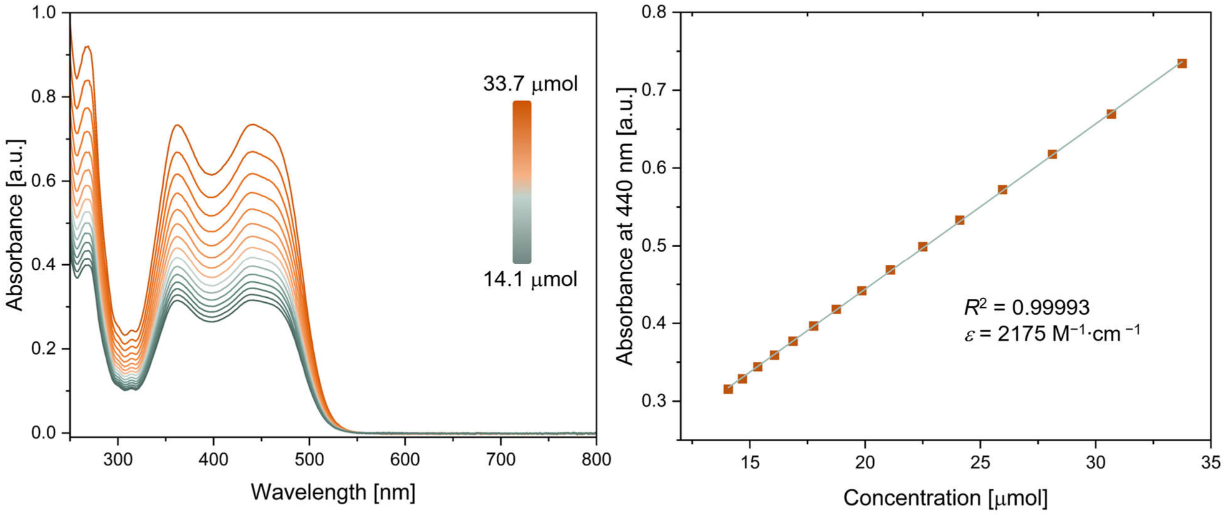

Figure S1. UV-vis spectra of the dilution series of (±)-**1-C** in 2-MeTHF at 298 K (left) and the derived Beer-Lambert plot for the absorbance at 440 nm (right) to exclude aggregation.

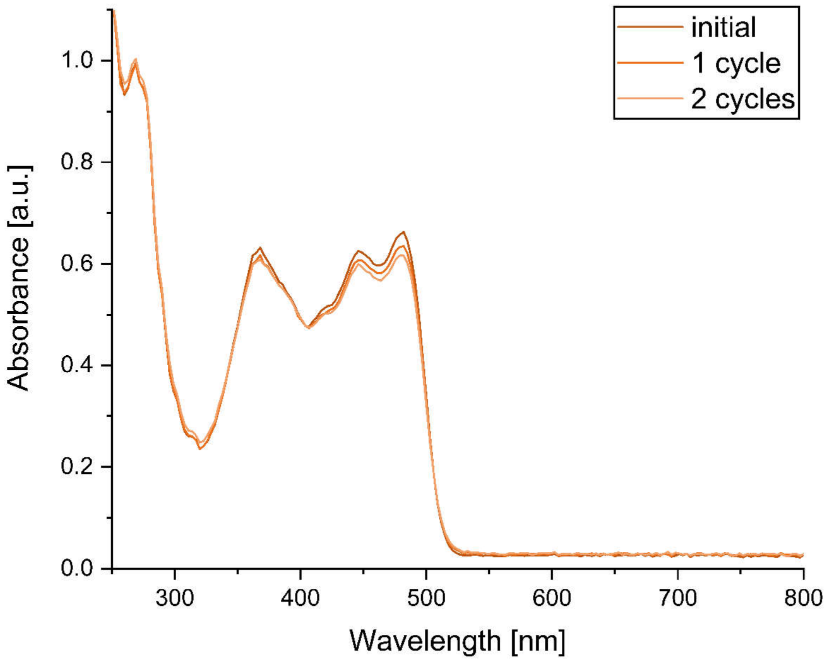

Figure S2. UV-vis absorbance spectra of (±)-**1-C** before irradiation and after one and two full cycles of 50 min irradiation 450 nm at 77 K, followed by warming up to 200 K and cooling back down to 77 K (c = 2.1·10^{-5} M in 2-MeTHF).

Irradiation of (±)-**1-C** at 298 K does not yield diradical (±)-**1-O** but only results in minor decomposition due to the strong irradiation. The spectral changes after irradiation are also not recovered thermally by leaving the sample in the dark for 1 h (Figure S3).

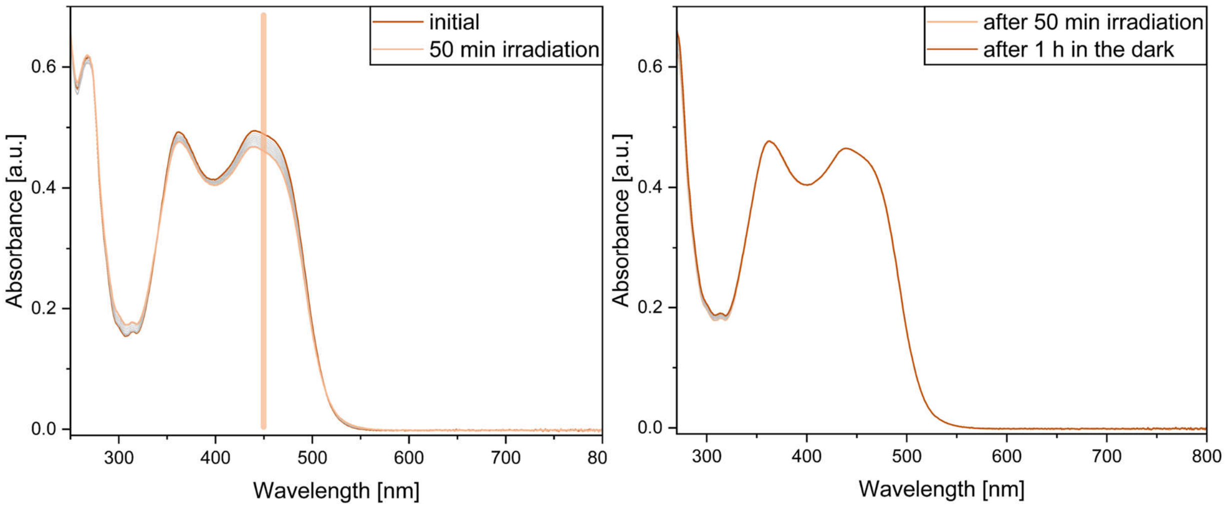

Figure S3. Irradiation of (±)-**1-C** monitored by UV-vis spectroscopy in 2-MeTHF with 450 nm light at 298 K for 50 min, showing slight decomposition (left). UV-vis spectra after leaving the previously irradiated sample in the dark for 50 min (right). $c = 2.2{\cdot}10^{-5}$ M.

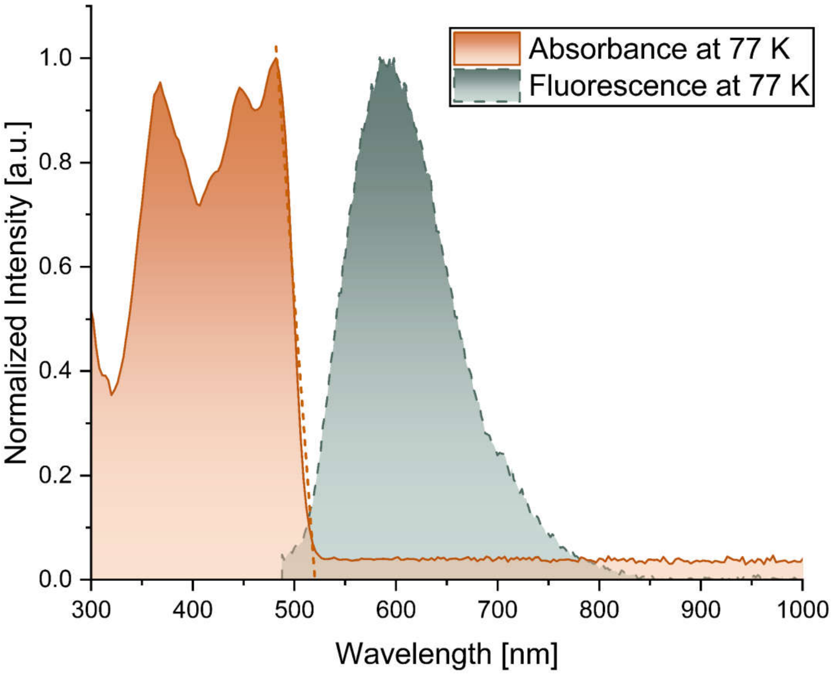

Figure S4. Absorbance (solid line) and emission (dashed line, excitation wavelength λ_{ex} = 479 nm) spectra of (±)-**1-C** with the linear fit λ_{onset} to estimate the optical band gap energy ΔE_{opt}.

The thermal back reaction from (±)-**1-O** to (±)-**1-C** can be followed by UV-vis spectrsocopy (Figure S5, top). Warming the irradiated sample to 110 K leas to the decay of the long wavelength band. The initial spectrum is however not regained. It is necessary to completely thaw the sample and cool it back to 77 K to regain the initial spectrum of

(±)-**1**-C (Figure S5, bottom). This suggests reversible structural or conformational changes occuring during the switching process.

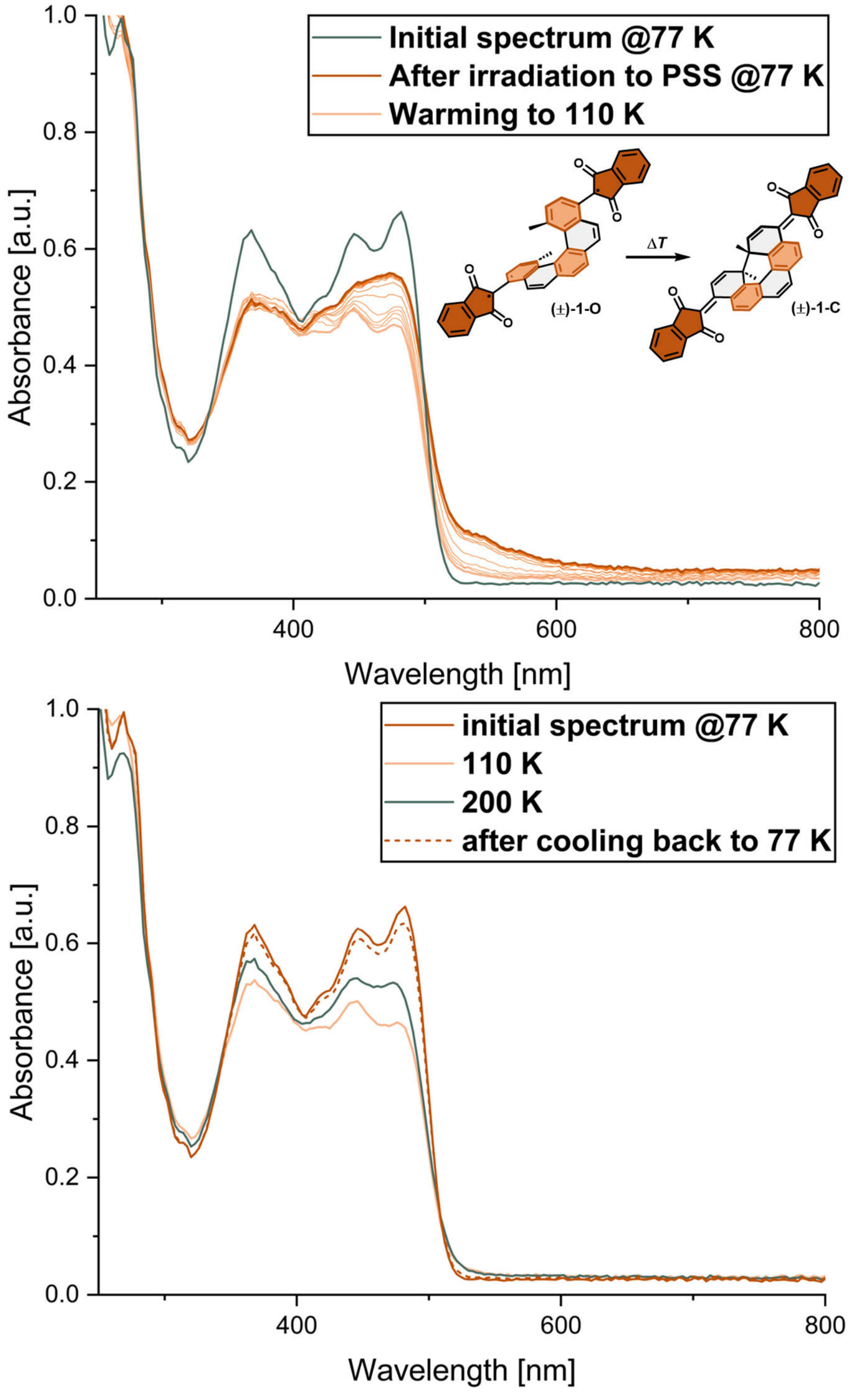

Figure S5. Top: Thermal back reaction of (±)-**1-O** after irradiating for 50 min at 77 K and warming to 110 K with 2 K·min^{-1}. Bottom: Further warming to 200 K and comparison with spectrum after cooling back down to 77 K (c = 2.1·10^{-5} M in 2-MeTHF)

S4. (VT)-EPR Spectroscopy

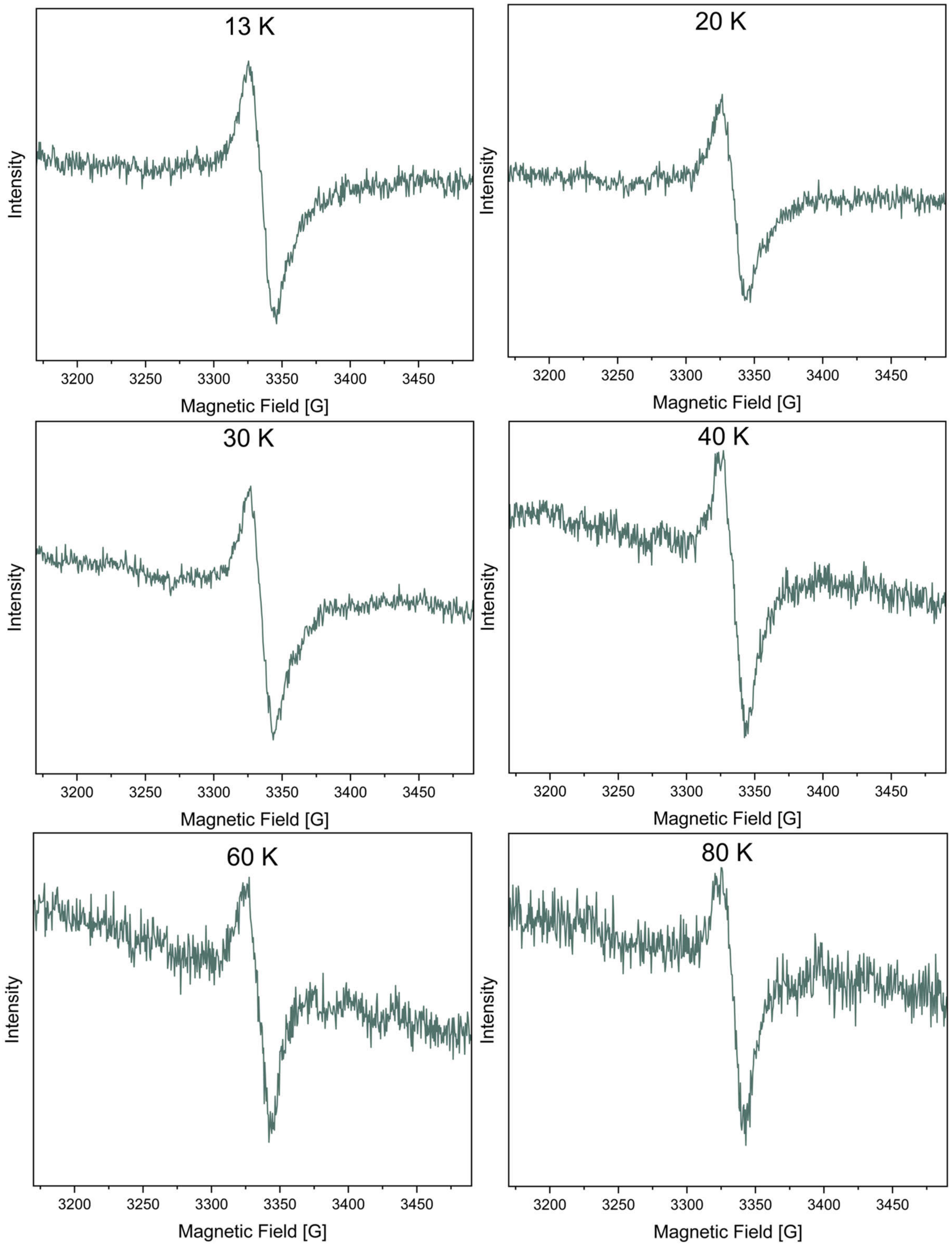

Figure S6. EPR spectra of (±)-**1-O** in toluene/CH_2Cl_2 1:1 at different temperatures. Experimental conditions: microwave frequency = 9.36 GHz; microwave powers = 0.01262 mW (13 K), 0.01262 mW (20 K), 0.05024 mW (30 K), 0.05024 mW (40 K), 0.05024 mW (60 K), 0.2 mW (80 K); modulation amplitude = 5 G.

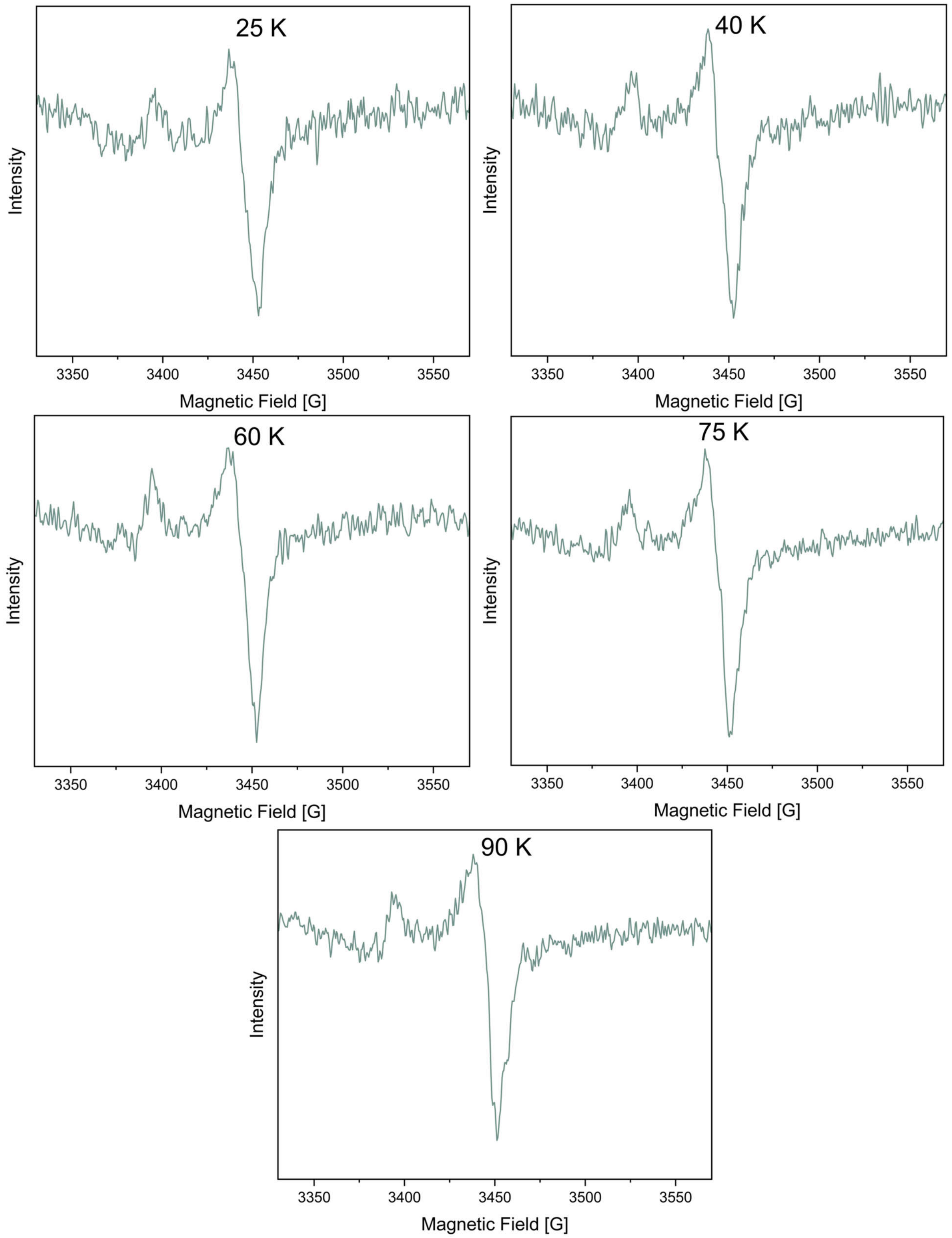

Figure S7. EPR spectra of (±)-**1**-**O** in 2-MeTHF at different temperatures. Experimental conditions: microwave frequency = 9.68 GHz; microwave power = 0.1543 mW for all temperatures; modulation amplitude = 5 G.

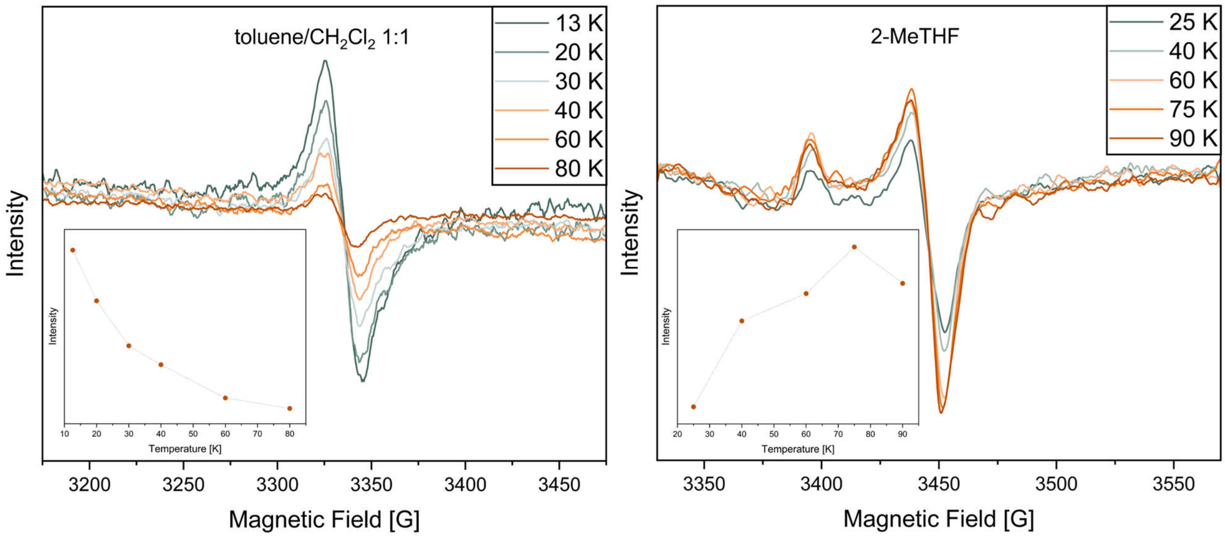

Figure S8. VT-EPR spectra of (±)-**1-O** in toluene/CH_2Cl_2 1:1 (left) and 2-MeTHF (right). Inset shows the peak intensity against the temperature.

To confirm the anticipated triplet spin state of (±)-**1-O** nutation experiments were conducted in 2-MeTHF at 80 K (Figure S9). The corresponding Fourier transformed nutation data revealed the expected nutation frequency of $\sqrt{2}\omega_0$ according to equation (1)[8–11]

$$\omega_{m_s,m_{s+1}} = \omega_0 \cdot \sqrt{S(S+1) - m_s(m_s+1)} \tag{1}$$

where m_s is the spin magnetic quantum number.

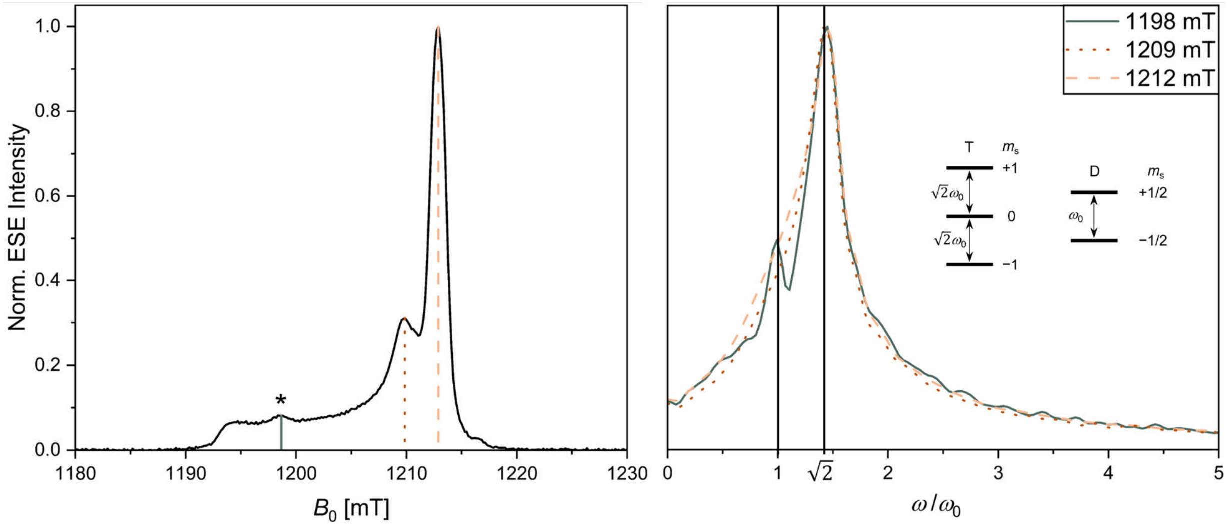

Figure S9. Nutation experiment of (±)-**1-O** generated from (±)-**1-C** after 5 h irradiation with a 450 nm pulsed laser in 2-MeTHF at 80 K (left). The star denotes the doublet glass artefact used as an internal reference. Fourier transformed nutation data at 1198 mT (solid line), 1209 mT (dotted line), and 1212 mT (dashed line). The first black bar denotes the doublet glass artefact the second bar the obtained peak at $\sqrt{2}$. Inset shows an illustration of the frequency relationship between doublet and triplet.

S5. Computational Details

All calculations were conducted with Gaussian 16 Revision A.03 on the chccs-cluster of the Department of Chemistry at Humboldt University of Berlin and on the bwForCluster JUSTUS 2 of the Department of Chemistry at the Albert Ludwigs University Freiburg. The structures are confirmed ground-state minima according to the analysis of their analytical frequencies computed at the same level, which show no imaginary frequencies. Unless otherwise stated exclusively the (*P*)-enantiomer for the open helicene or the (*S*,*S*)-enantiomer for the closed *semi*-quinoidal molecule were calculated. All geometry optimizations as closed-shell singlets (CS) or as triplets (T) were performed at the DFT:D3[12]-B3LYP/6-31G(d,p)[13] level of theory. In order to describe the molecules (±)-**1** as open-shell singlet's (OS) the guess=mix keyword was used to create as initial wave function a 1:1 mixture of singlet and triplet states with a squared spin expectation value $\langle S^2 \rangle = 1$. The final energies were calculated at the DFT-D3/UB3LYP[14,15]/def2-TZVP level of theory. The time-dependent density-functional theory (TD-DFT) calculations were performed on the geometry-optimized structures at the TD-DFT:D3-UCAM-B3LYP/def2-TZVP[16] level of theory incorporating 20 states. The conductor-like polarizable continuum model (CPCM)[17] was used as solvent model with dimethylformamide.

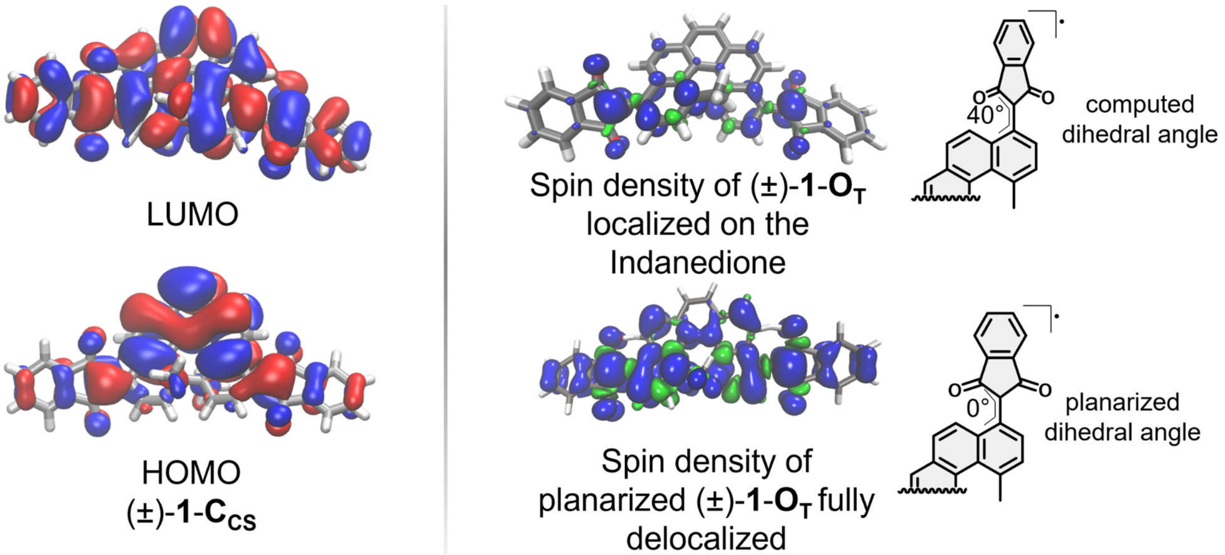

Figure S10. HOMO and LUMO representations (isovalue = ±0.009) of (±)-**1**-**C** as closed-shell singlet as well as spin density (difference between α and β densities) representation (isovalue = ±0.0025) of (±)-**1**-**O** as triplet in comparison with a forcefully planarized geometry of (±)-**1**-**O**. Only the (*P*)- or (*S*,*S*)-enantiomer are shown for clarity.

S6. (Spectro)electrochemistry

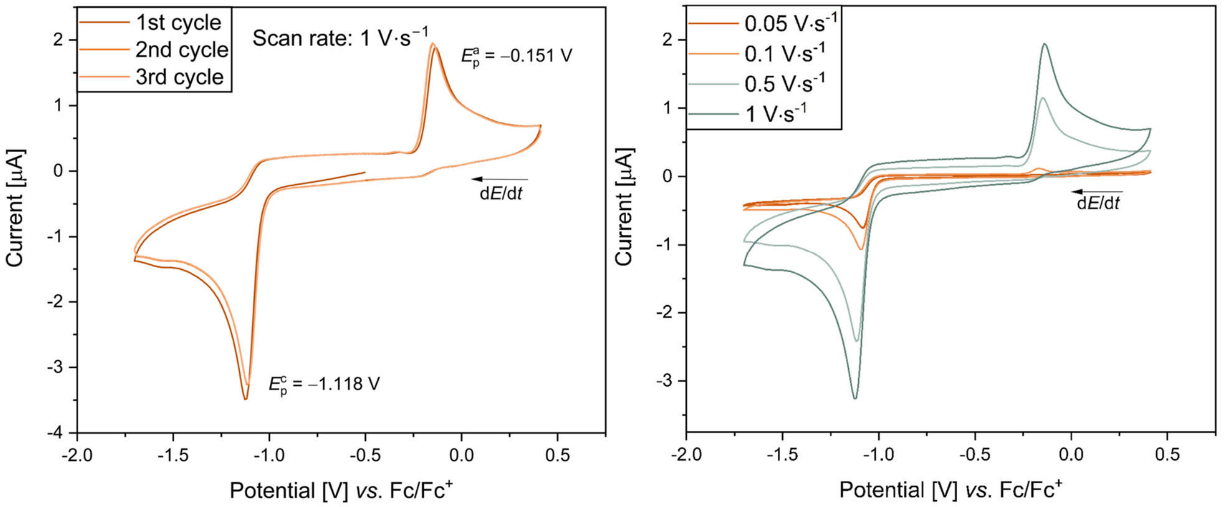

Figure S11. Cyclic voltammograms of (±)-1-C over several cycles (left) and at scan rates between (0.05–1) V·s^{-1} (c = 0.5 mM with 0.1 M nBuN$_4$PF$_6$ in DMF)

By measuring cyclic voltammetry and determination of the half-wave potential of (±)-**1**-**C**, the reduction potential (E_{red}) and oxidation potential (E_{ox}) can be derived and from there on the HOMO energy. According to *Koopman's* theorem, the first ionization energy is equivalent to the negative HOMO energy in closed-shell Hartree–Fock theory.[18] Pommerehne and co-workers suggested an offset of 4.8 eV as the ionization potential of ferrocene on the vacuum scale.[19] According to equation (2) the HOMO energy can be calculated.

$$E_{\mathrm{HOMO}} = -e \cdot E_{\mathrm{ox}} - 4.8\ \mathrm{eV} \qquad (2)$$

With the HOMO energy E_{HOMO}, and the experimentally obtained optical gap ΔE_{opt}, the LUMO energy E_{LUMO} can be accessed according to equation (3).

$$E_{\mathrm{LUMO}} = E_{\mathrm{HOMO}} + \Delta E_{\mathrm{opt}} \qquad (3)$$

A sufficient agreement of experimentally determined energy levels and energy differences with the calculated values was found for (±)-**1**-**C** (Table S1).

Table S1. Comparison of the band gap (ΔE_{opt}) of (±)-**1-C** by experimentally determined with theoretically calculated values for HOMO and LUMO. The calculated values for HOMO and LUMO are computed at the DFT-UB3LYP/def2-TZVP level of theory and marked with the subscript "calc".

λ_{onset} [nm]	ΔE_{opt} [eV]	E_{HOMO} [eV]	E_{LUMO} [eV]	ΔE_{calc} [eV]	$E_{HOMO,calc}$ [eV]	$E_{LUMO,calc}$ [eV]
519	2.39	–6.07	–3.68	3.04	–6.22	–3.18

The following table lists important spectroscopic and electrochemical properties of the herein reported (±)-**1-C** and our previously reported [5]helicene diketone and bis(dicyanomethylidene).[20]

Table S2. Comparison of the optical band gap (ΔE_{opt}), the experimentally determined HOMO and LUMO energy levels (E_{HOMO}, E_{LUMO}) and the cathodic and anodic potentials (E_p^c, E_p^a) of (±)-**1-C** with reported diketone and bis(dicyanomethylidene).

	ΔE_{opt} [eV]	E_{HOMO} [eV]	E_{LUMO} [eV]	E_p^c [V]	E_p^a [V]
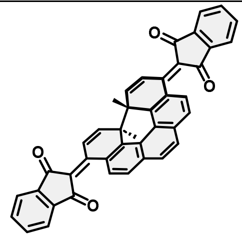	2.39	–6.07	–3.68	–1.118	–0.151
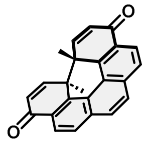	3.28	–6.47	–3.19	–2.001<br>–2.270	–0.768
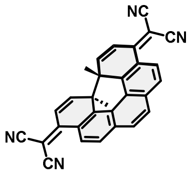	2.78	–6.45	–3.67	–1.369	–0.295

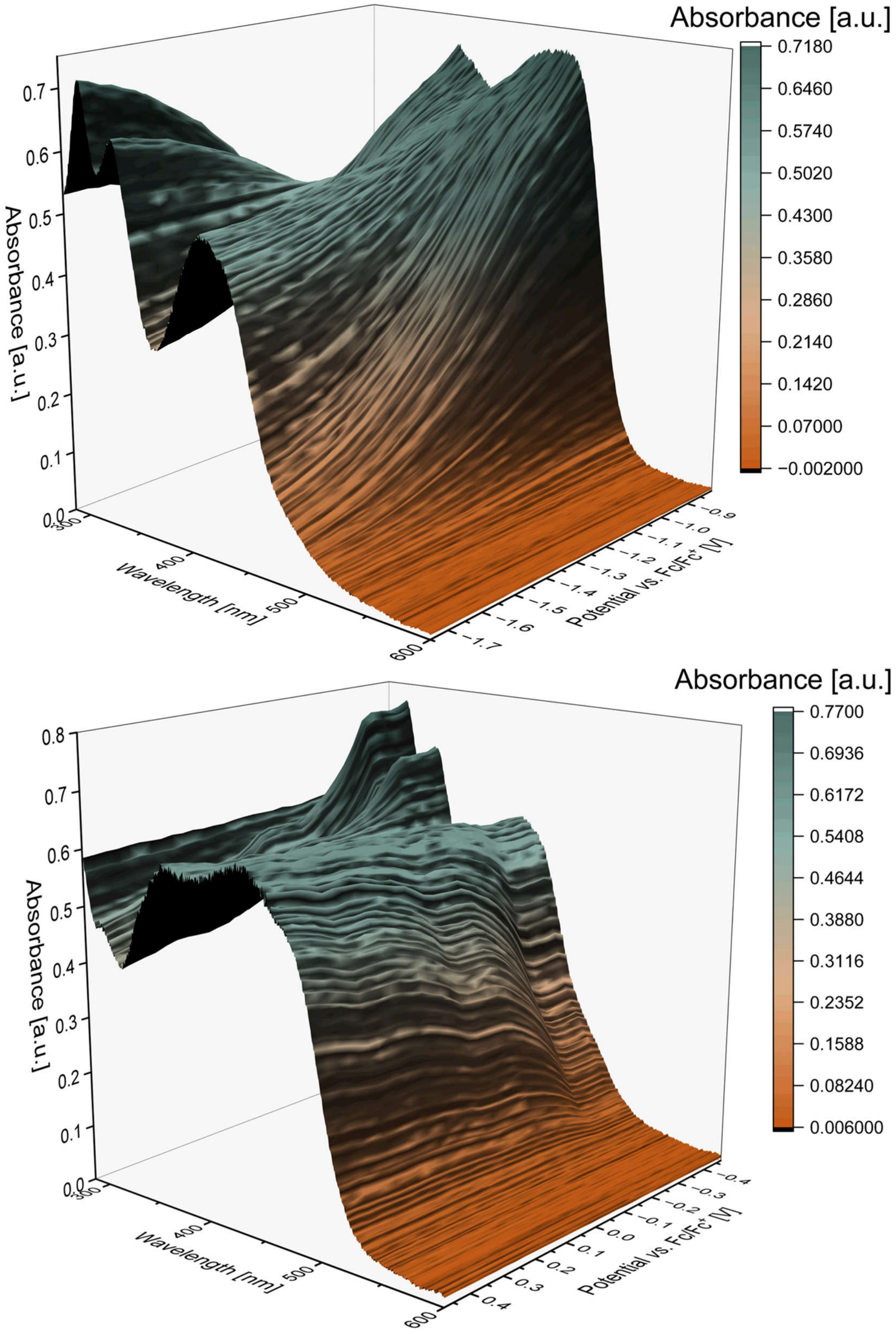

Figure S12. Spectroelectrochemistry of (±)-**1**-**C**. Plotted is the absorbance, the wavelength and the applied potential during reduction (top) and oxidation (bottom). The experiment was carried out in a thin layer quartz glass spectroelectrochemical cell (*d* = 1 mm) in DMF with 0.1 M Bu_4NPF_6, platinum mesh electrode, *c* = 0.3 mM, d*E*/d*t* = 10 mV s−1, T = 295 K.

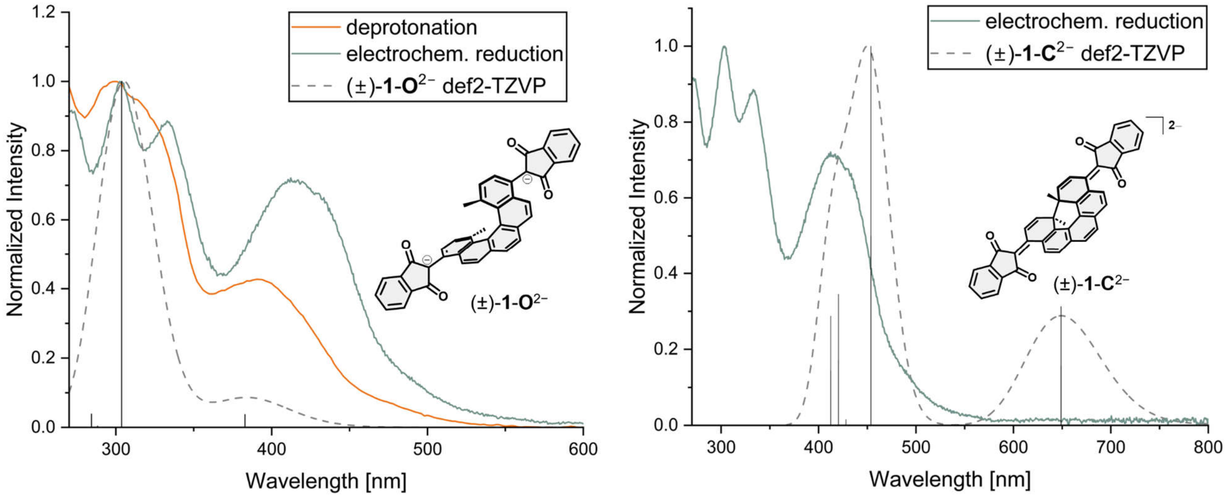

Figure S13. Calculated UV-vis spectrum of (±)-**1**-**O**$^{2–}$ (left) and (±)-**1**-**C**$^{2–}$ (right) together with the experimentally generated bis anions *via* electrochemical reduction (c = 0.3 mM in DMF) or deprotonation (c = $2.3\cdot10^{-5}$ M in THF). Level of theory TD-DFT:D3-UCAM-B3LYP/def2-TZVP/CPCM(DMF). Calculated spectra are shown with a line width fitting parameter σ = 0.16 eV, scaling factor of 9.97, and –90 nm UV correction. The similarity factor for (±)-**1**-**O**$^{2–}$ is 95%.

S7. Crystallographic Data

X-ray Crystal Structure of (±)-1-C

Orange crystals were grown by slow evaporation from $CDCl_3$ at 20 °C.

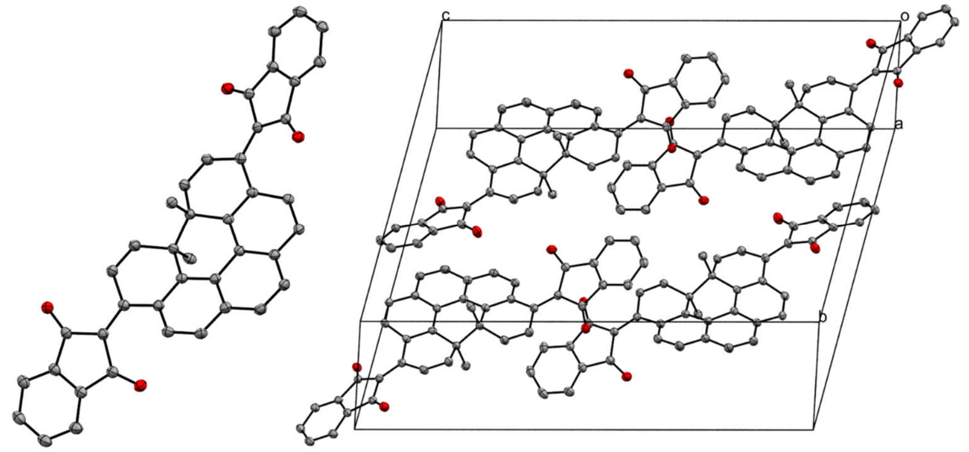

Figure S14.- X-ray structure of (±)-**1-C** at 100 K. Ellipsoids are shown at 50% probability, hydrogens are omitted for clarity. Color code: carbon, grey; oxygen, red.

Table S3. Crystal data and structure refinement for (±)-**1-C**.

CCDC number	2289901
Empirical formula	$C_{173}H_{98}Cl_{15}O_{16}$
Formula weight	2964.26
Temperature/K	100.00
Crystal system	triclinic
Space group	*P*-1
a/Å	7.7592(8)
b/Å	18.918(2)
c/Å	23.136(2)
α/°	80.552(4)
β/°	84.145(4)
γ/°	80.771(4)
Volume/Å^3	3296.7(6)
Z	1
ρ_{calc}/ g/cm^3	1.493
μ/mm^{-1}	0.386
F(000)	1519.0
Crystal size/mm^3	0.21 × 0.2 × 0.11
Radiation	MoKα (λ = 0.71073)
2Θ range for data collection/°	3.912 to 53.118
Index ranges	$-9 \leq h \leq 9$, $-23 \leq k \leq 23$, $-29 \leq l \leq 29$
Reflections collected	117640
Independent reflections	13628 [R_{int} = 0.0364, R_{sigma} = 0.0187]
Data/restraints/parameters	13628/3/966
Goodness-of-fit on F^2	1.026
Final *R* indexes [$I \geq 2\sigma(I)$]	R_1 = 0.0469, wR_2 = 0.1190
Final *R* indexes [all data]	R_1 = 0.0560, wR_2 = 0.1264
Largest diff. peak/hole / e Å^{-3}	0.80/–0.67

A close intermolecular packing with antiparallel aligned dipoles is observed with four molecules occupying a unit cell. Each terminal cyclohexadiene ring of the closed [5]helicene core interacts with the indanedione benzene ring of a neighboring molecule through π–π-stacking, resulting in a short $C_{Carbonyl}$–$C_{Carbonyl}$ distance of 3.37 Å. Additionally, a close interaction between the indanedione benzene ring and the central [5]helicene benzene ring is visible in the molecular packing. The helical pitch angle along the inner sp^2-hybridized C−C−C−C atoms is 15.00°, with a nearly axial displacement of the methyl groups. The alternating bond lengths of the terminal cyclohexadiene clearly indicate a quinoidal structure.

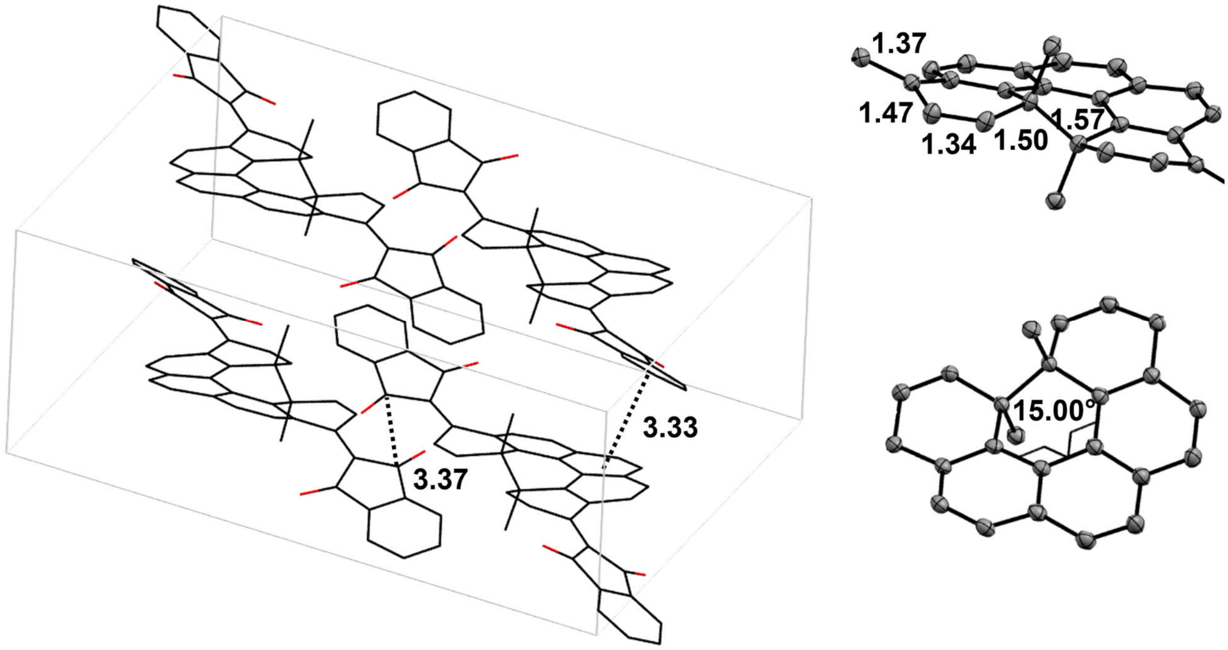

Figure S15. Crystal packing in the unit cell of (±)-**1**-**C** (left) and helical core with bond lengths (in Å) and helical pitch angle (right). Structure was measured at 100 K. Ellipsoids are shown at 50% probability, hydrogens are omitted for clarity. Color code: carbon, grey; oxygen, red.

X-ray Crystal Structure of (±)-1-$O^{2-}(Et_3NH^+)_2$

Orange crystals were grown by slow evaporation from toluene/$CHCl_3$ at 20 °C.

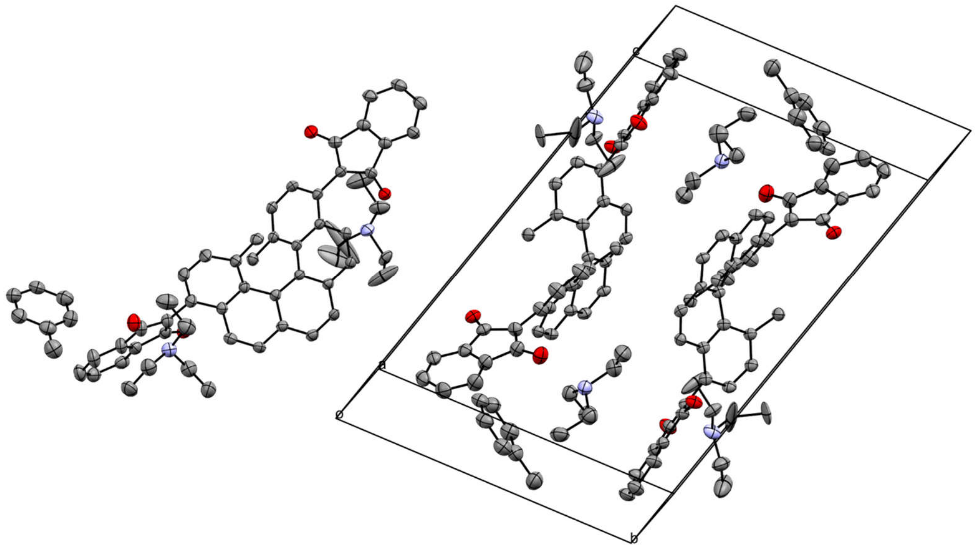

Figure S16. X-ray structure of (±)-**1-O**$^{2-}$(Et_3NH^+)$_2$ at 100 K. Ellipsoids are shown at 50% probability, hydrogens are omitted for clarity. Color code: carbon, grey; oxygen, red; nitrogen, blue.

Table S4. Crystal data and structure refinement for (±)-**1-O**$^{2-}$(Et_3NH^+)$_2$.

CCDC number	2452566
Empirical formula	$C_{122}H_{116}N_4O_{10}$
Formula weight	1798.18
Temperature/K	100.00
Crystal system	triclinic
Space group	*P*-1
a/Å	9.242(4)
b/Å	13.893(5)
c/Å	20.548(8)
α/°	75.611(13)
β/°	83.427(14)
γ/°	77.356(13)
Volume/Å^3	2488.5(17)
Z	1
ρ_{calc}/ g/cm^3	1.200
μ/mm^{-1}	0.075
F(000)	956.0
Crystal size/mm^3	Not determined
Radiation	MoKα (λ = 0.71073)
2Θ range for data collection/°	4.082 to 61.474
Index ranges	$-13 \le h \le 13$, $-19 \le k \le 19$, $-29 \le l \le 29$
Reflections collected	156251
Independent reflections	15371 [R_{int} = 0.1375, R_{sigma} = 0.0861]
Data/restraints/parameters	15371/37/693
Goodness-of-fit on F^2	1.021
Final *R* indexes [$I \ge 2\sigma(I)$]	R_1 = 0.1262, wR_2 = 0.3118
Final *R* indexes [all data]	R_1 = 0.2062, wR_2 = 0.3725
Largest diff. peak/hole / e Å^{-3}	1.65/–0.62

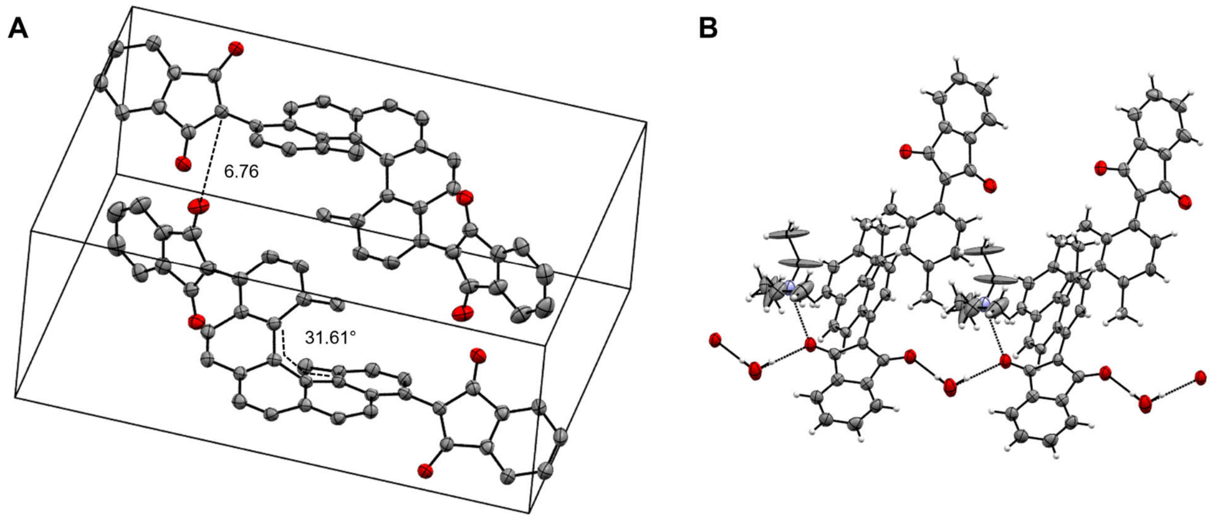

Figure S17. A) Crystal packing of (±)-**1**-$\mathbf{O^{2-}}$(Et_3NH^+)$_2$ with helical pitch (solvent and counter ion omitted for clarity); B) Side view on the chain-like hydrogen bonding between the indanedione and crystal water. Ellipsoids are shown at 50% probability, hydrogens are omitted for clarity. Color code: carbon, grey; oxygen, red; nitrogen, blue.

S8. Selected NMR Spectra

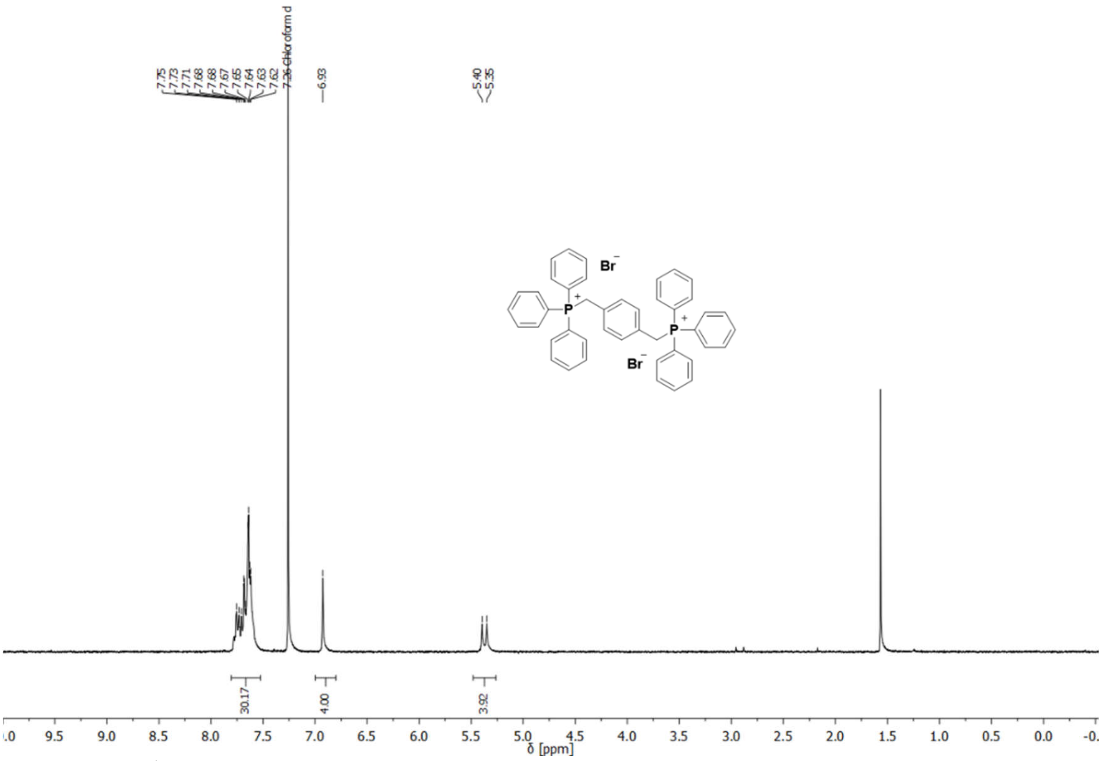

Figure S18. ^{1}H NMR spectrum (500 MHz, $CDCl_3$, 25 °C) of **3**.

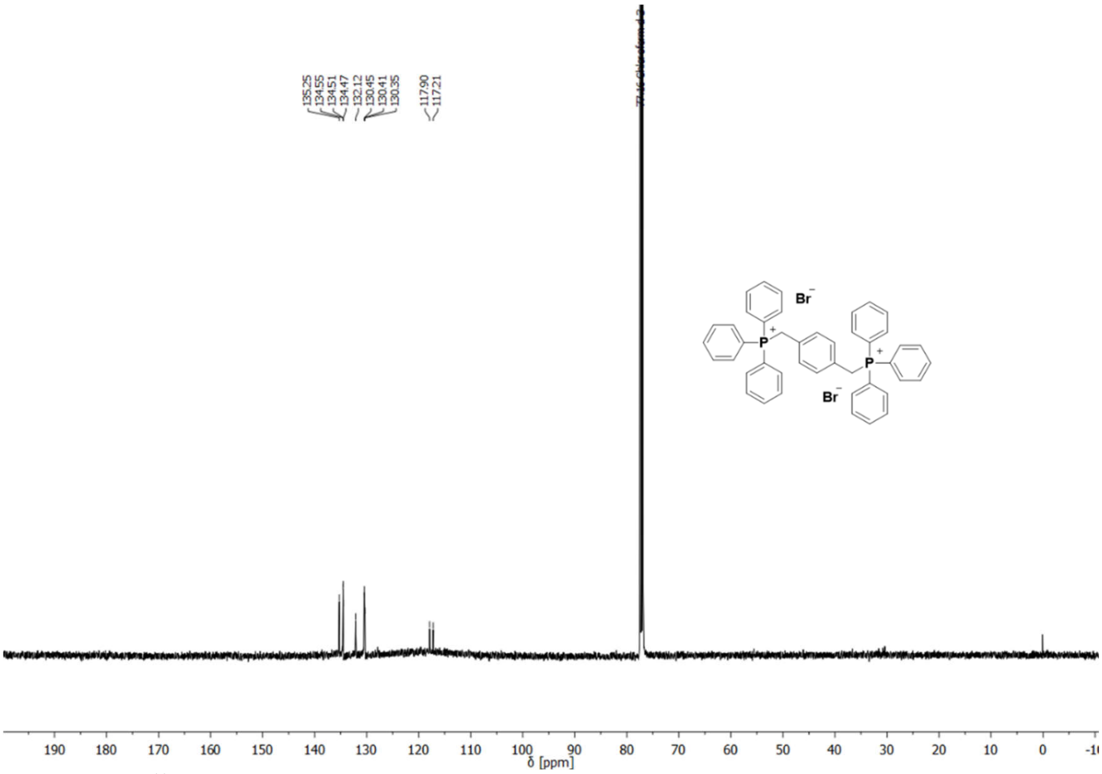

Figure S19. ^{13}C NMR spectrum (126 MHz, $CDCl_3$, 25 °C) of **3**.

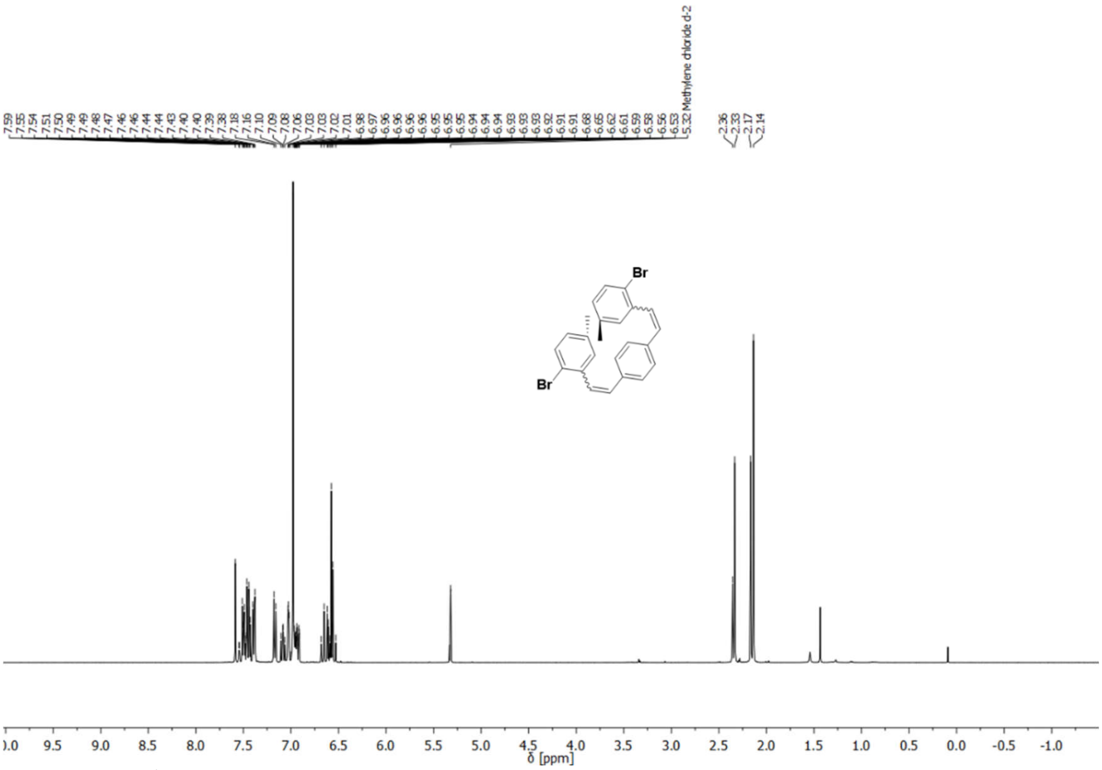

Figure S20. ^{1}H NMR spectrum (500 MHz, $CDCl_3$, 25 °C) of **4.**

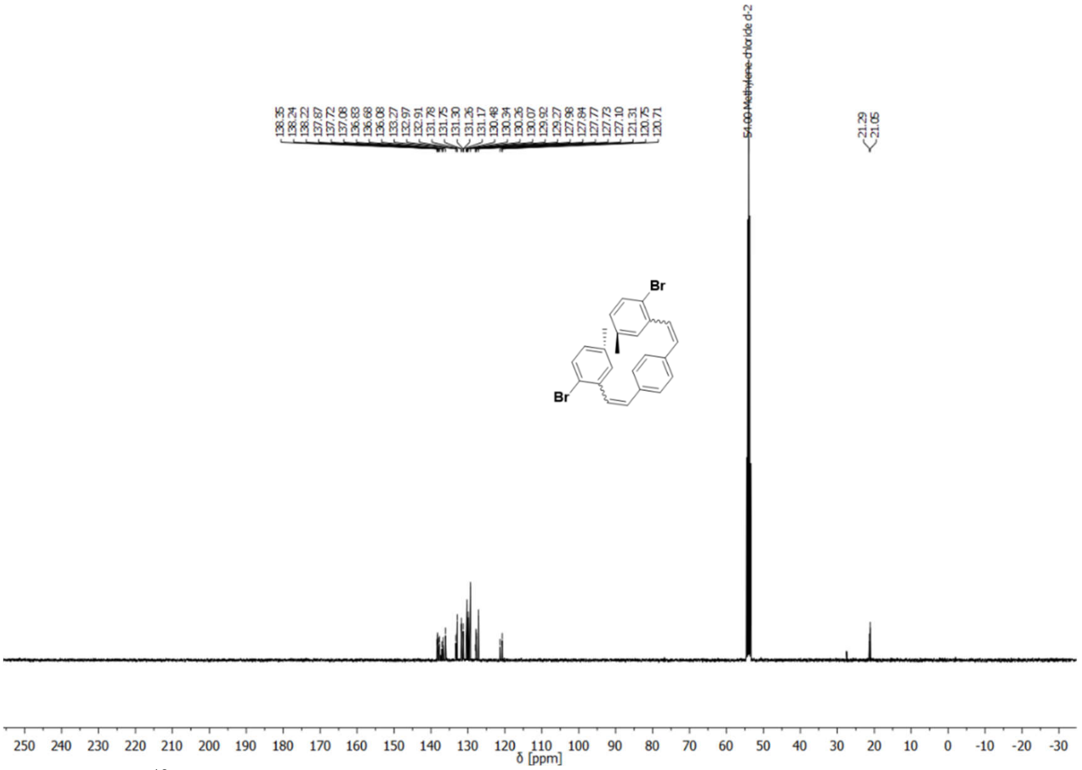

Figure S21. ^{13}C NMR spectrum (126 MHz, $CDCl_3$, 25 °C) of **4.**

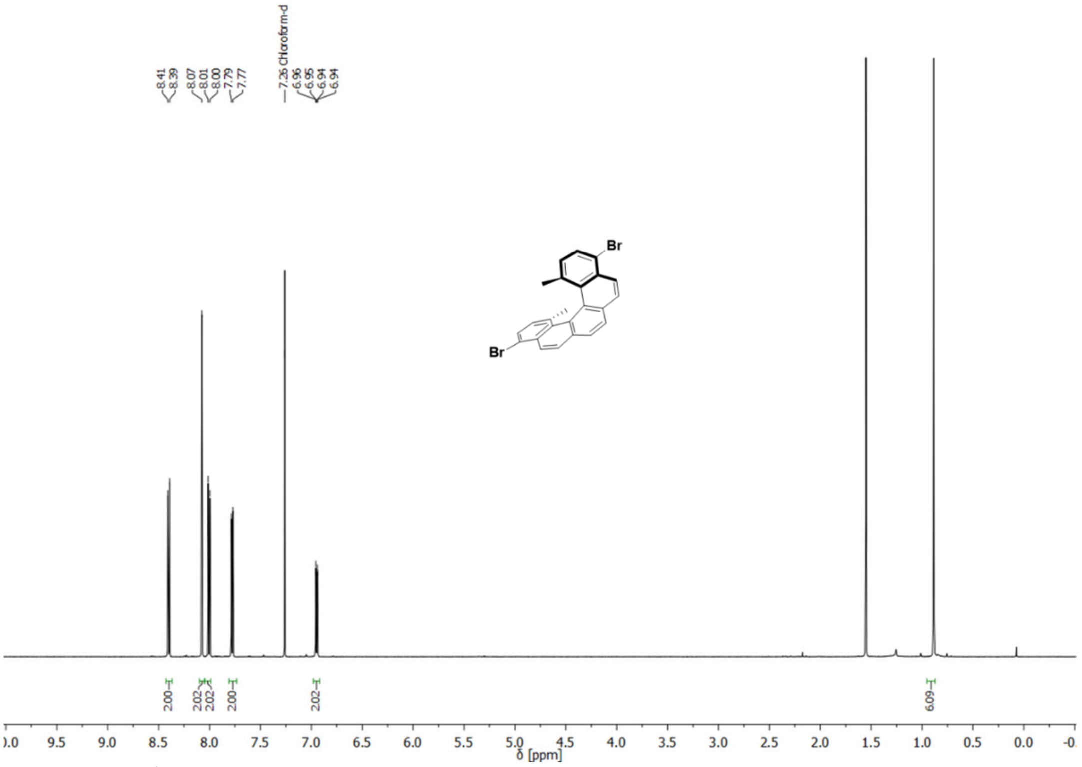

Figure S22. ^{1}H NMR spectrum (500 MHz, $CDCl_3$, 25 °C) of (±)-**2**.

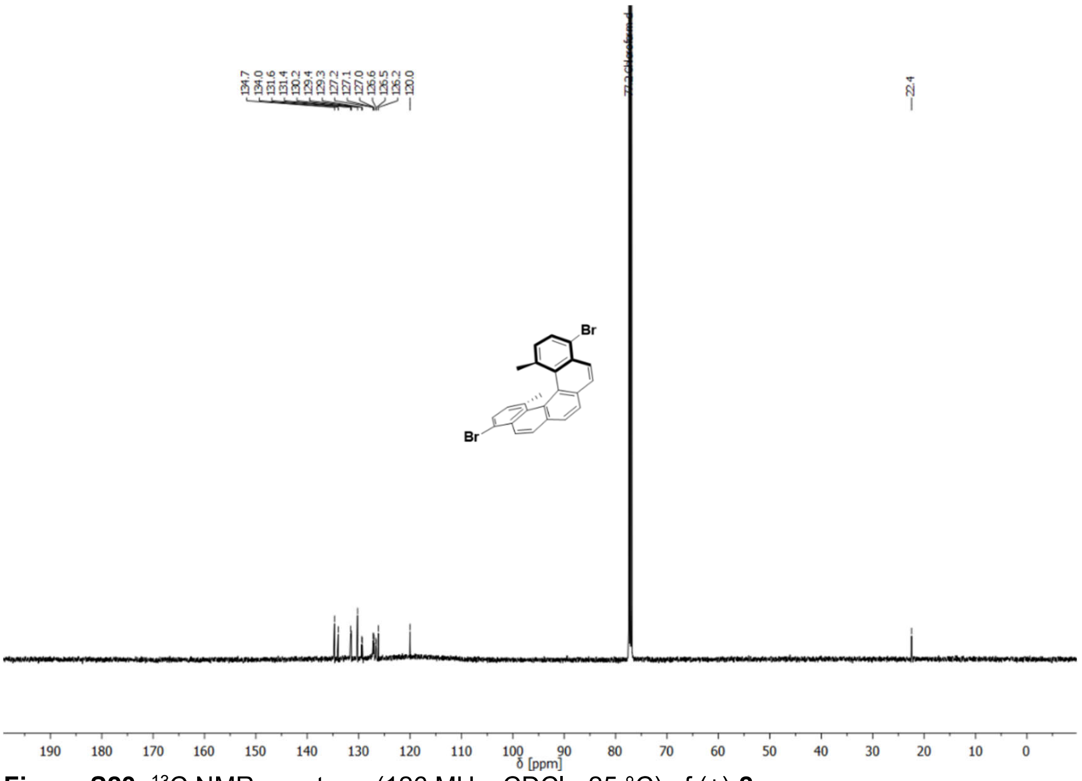

Figure S23. ^{13}C NMR spectrum (126 MHz, $CDCl_3$, 25 °C) of (±)-**2**.

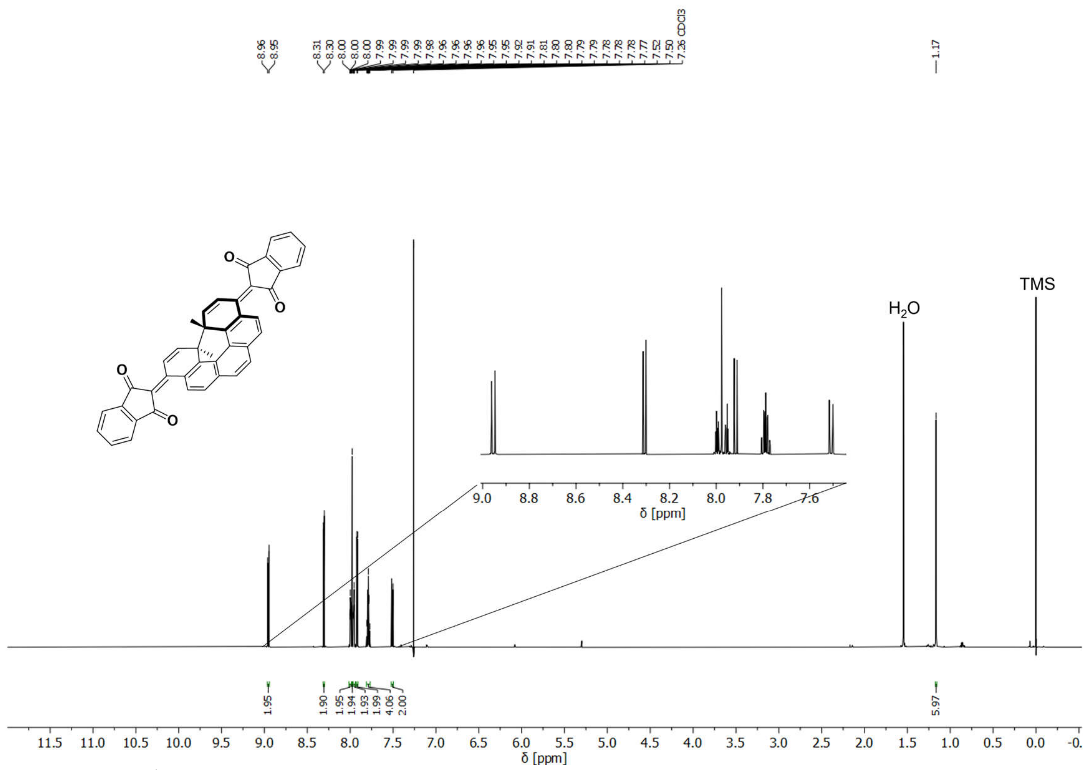

Figure S24. ^{1}H NMR spectrum (500 MHz, $CDCl_3$, 25 °C) of (±)-**1-C**.

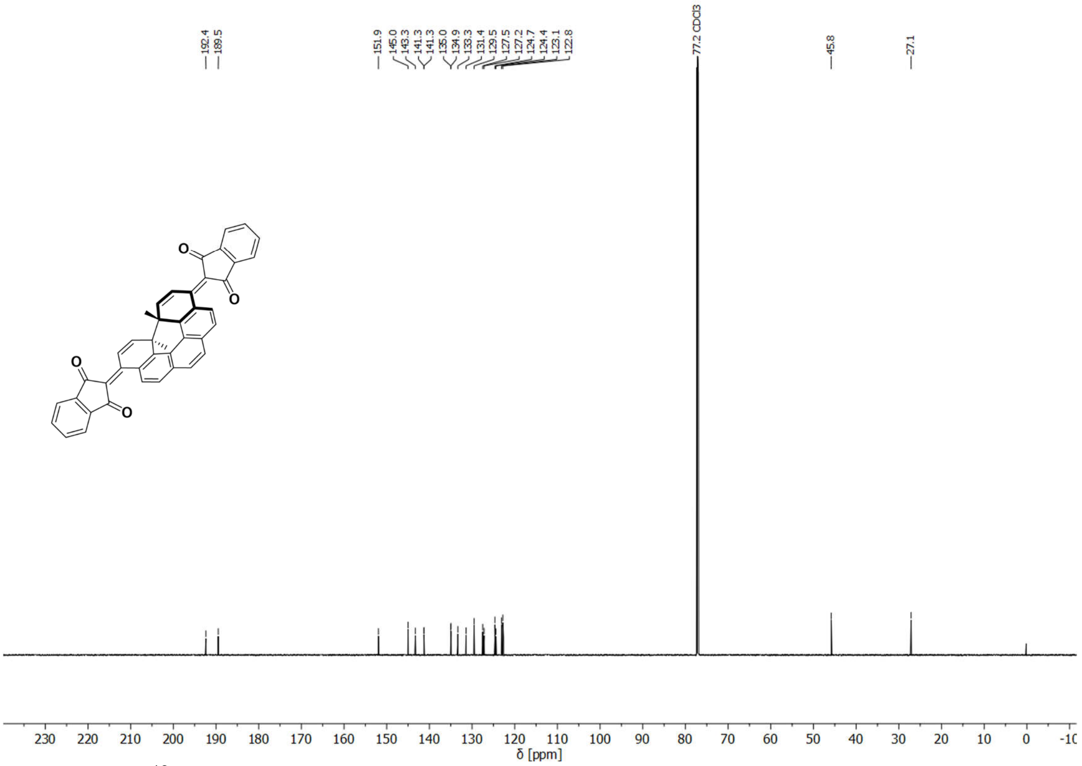

Figure S25. ^{13}C NMR spectrum (126 MHz, $CDCl_3$, 25 °C) of (±)-**1-C**.

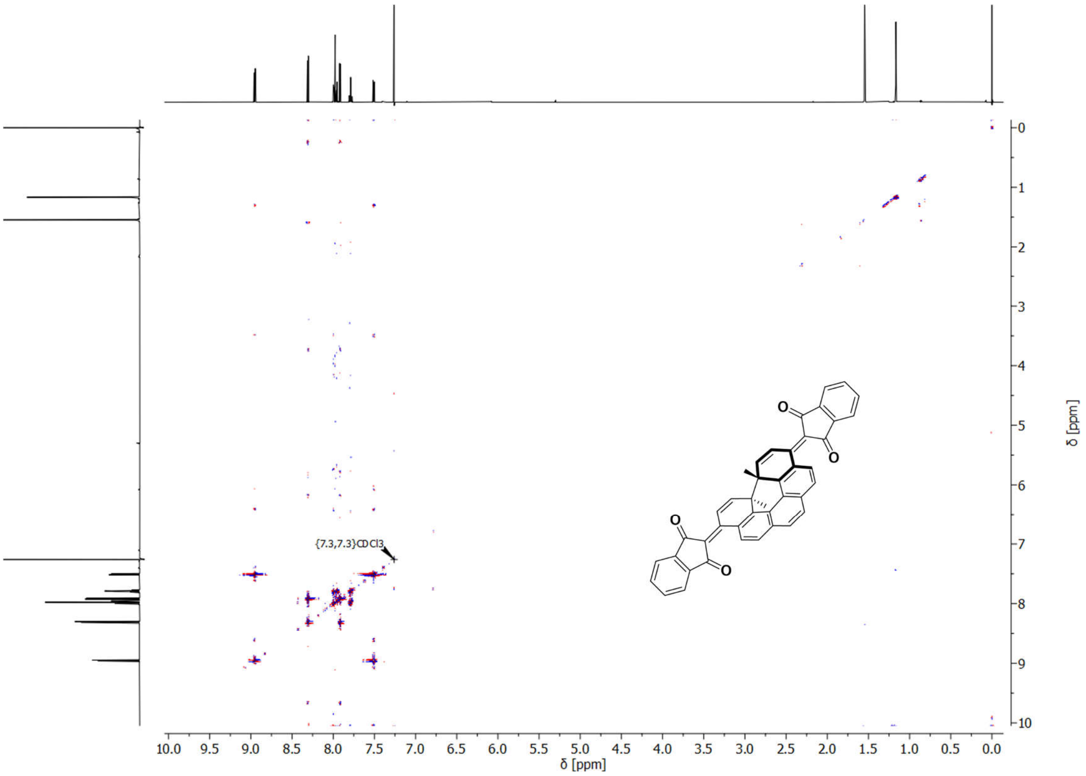

Figure S26. $^{1}H,^{1}H$-COSY spectrum (500 MHz, $CDCl_3$, 25 °C) of (±)-**1-C**.

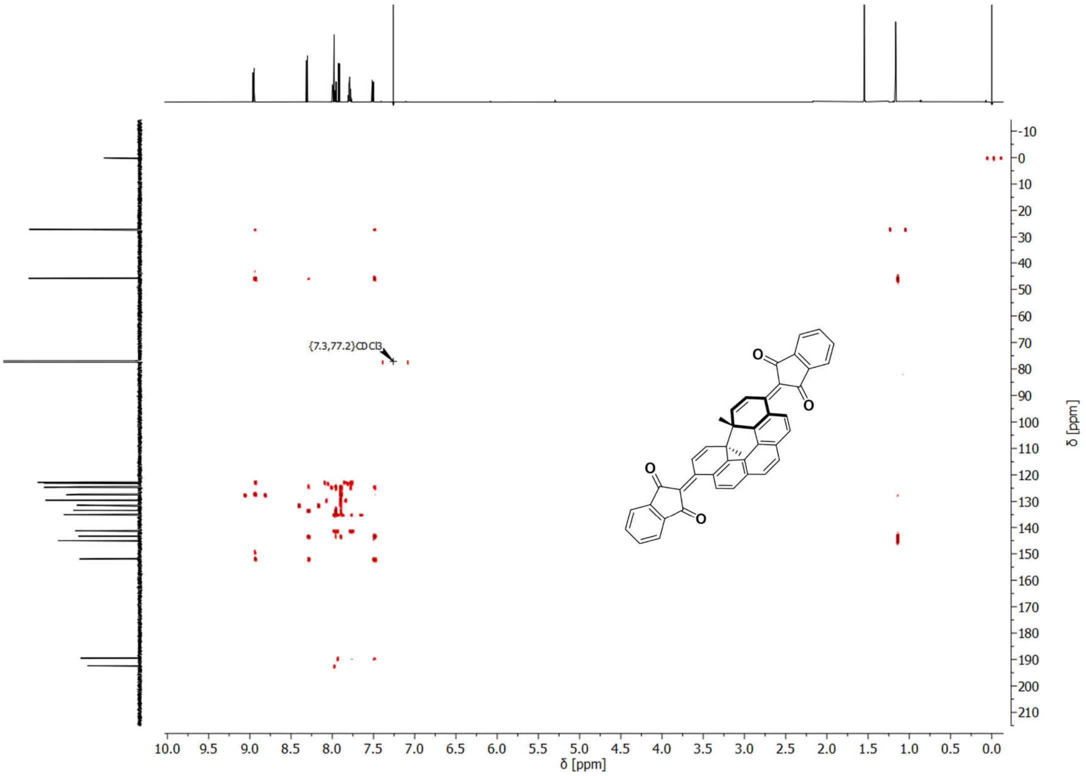

Figure S27. $^{1}H,^{13}C$-HSQC spectrum (500 MHz, 126 MHz, $CDCl_3$, 25 °C) of (±)-**1-C**.

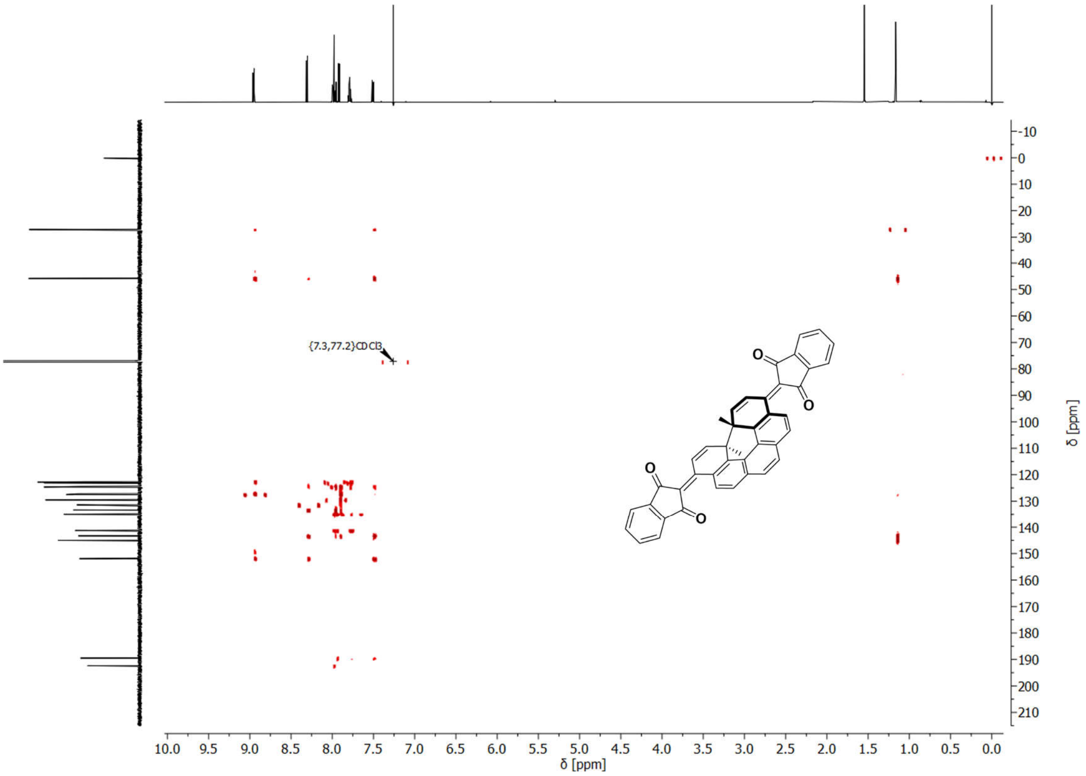

Figure S 28. $^{1}H,^{13}C$-HMBC spectrum (500 MHz, 126 MHz, $CDCl_3$, 25 °C) of (±)-**1-C**.

For (±)-**1-O-H₂** the keto-form dominates over the enol-form. A ^{1}J C–H coupling for the acidic proton at 5.13 ppm is clearly visible in the $^{1}H,^{13}C$-HSQC spectrum which suggests that this proton is bound to a carbon (Figure S26, inset right). Additionally, two highly down-field shifted ^{13}C signals can be observed in the ^{13}C NMR spectrum which can be assigned to the rotationally restricted ketones of the indanedione subunit (Figure S29, inset left).

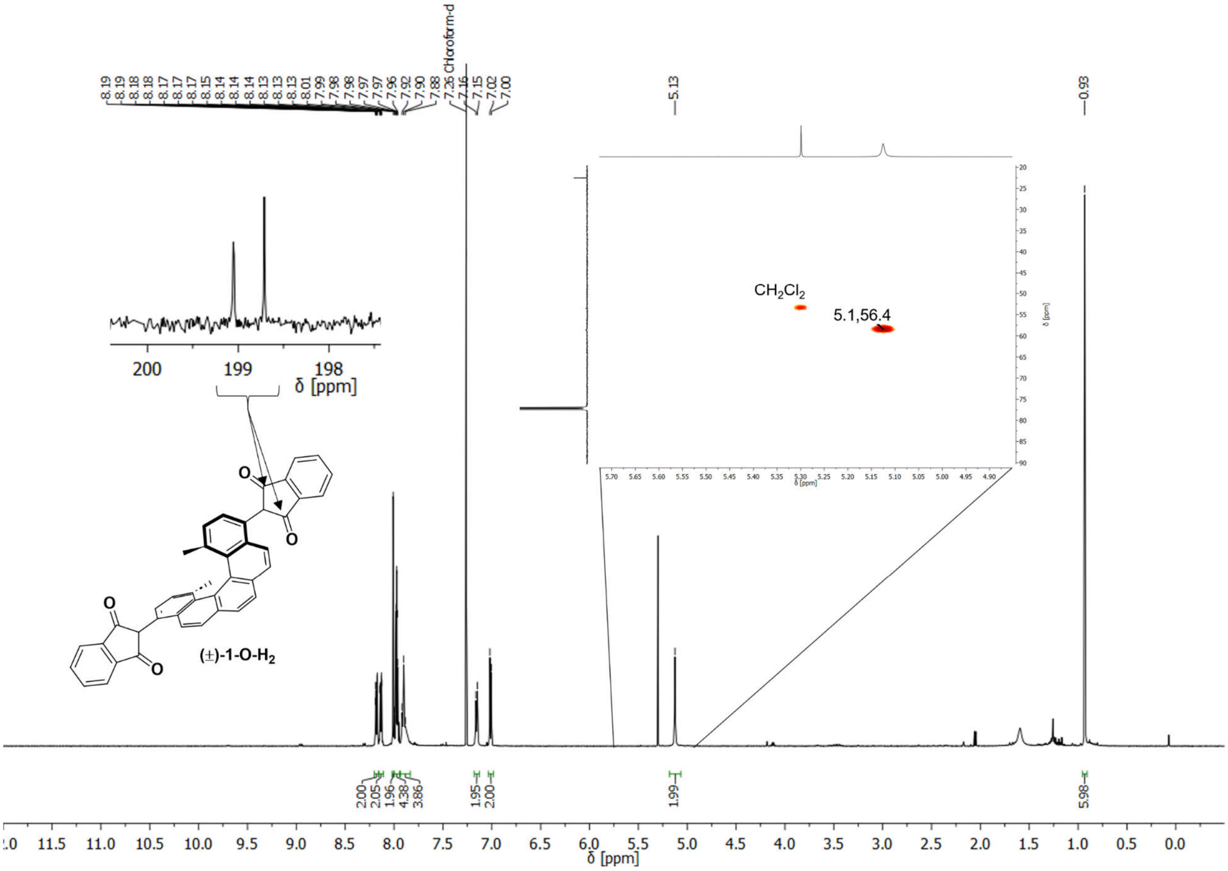

Figure S29. ^1H NMR spectrum (500 MHz, $CDCl_3$, 25 °C) of (±)-**1-O-H2**, inset right shows zoom of the ^1H,^{13}C-HSQC spectrum (500 MHz, 126 MHz, $CDCl_3$, 25 °C) of (±)-**1-O-H2**. Inset (left) shows a zoomed view on the ^{13}C NMR spectrum (126 MHz, $CDCl_3$, 25 °C) of (±)-**1-O-H2**.

The deprotonation of (±)-**1-O-H2** to (±)-**1-O**$^{2-}$ can also be followed by ^1H NMR with most significant change being the loss of the signal of the acidic sp^3 C–H signal at 5.13 ppm (Figure S30).

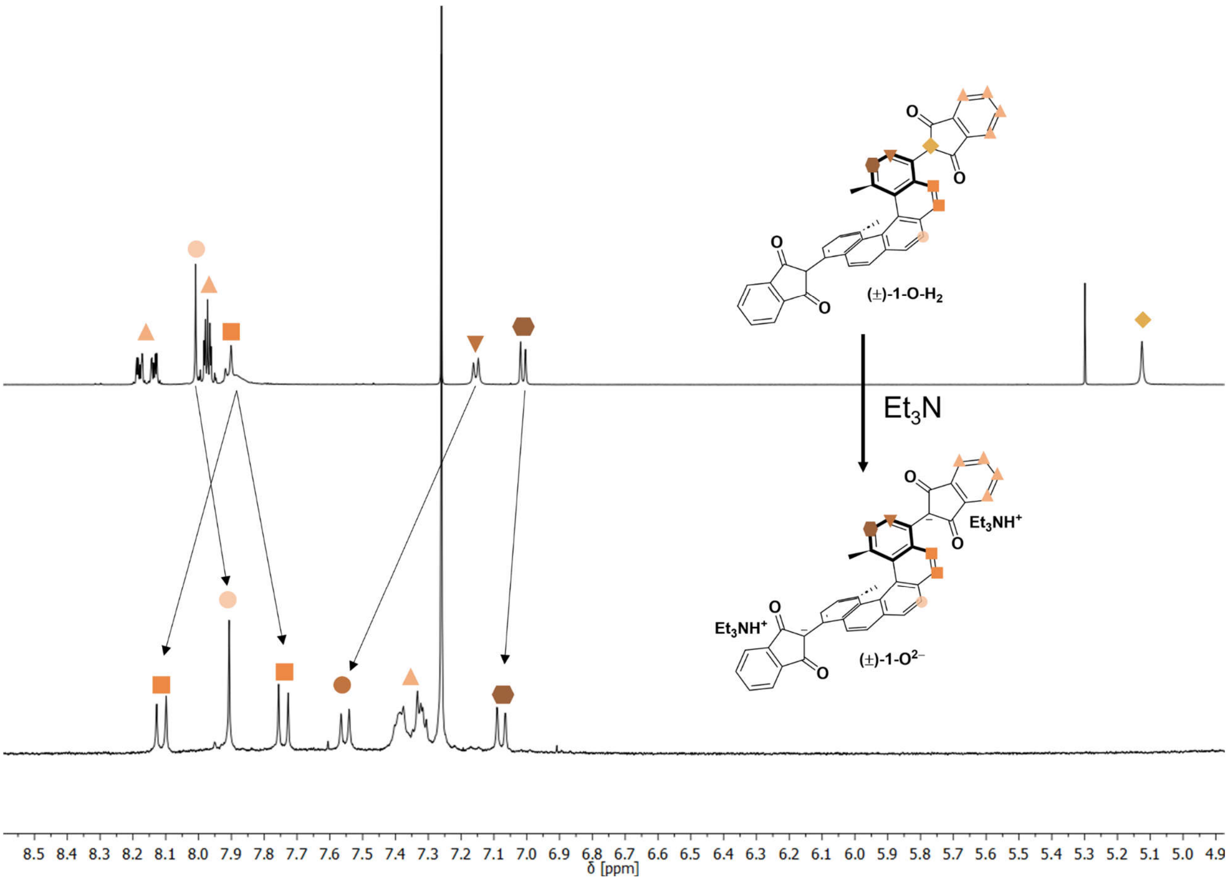

Figure S30. ^{1}H NMR spectrum (500 MHz, $CDCl_3$, 25 °C) of (±)-**1-O-H$_2$** (top) and (±)-**1-O^{2-}** (bottom).

S9. Selected ATR-FT-IR Spectra

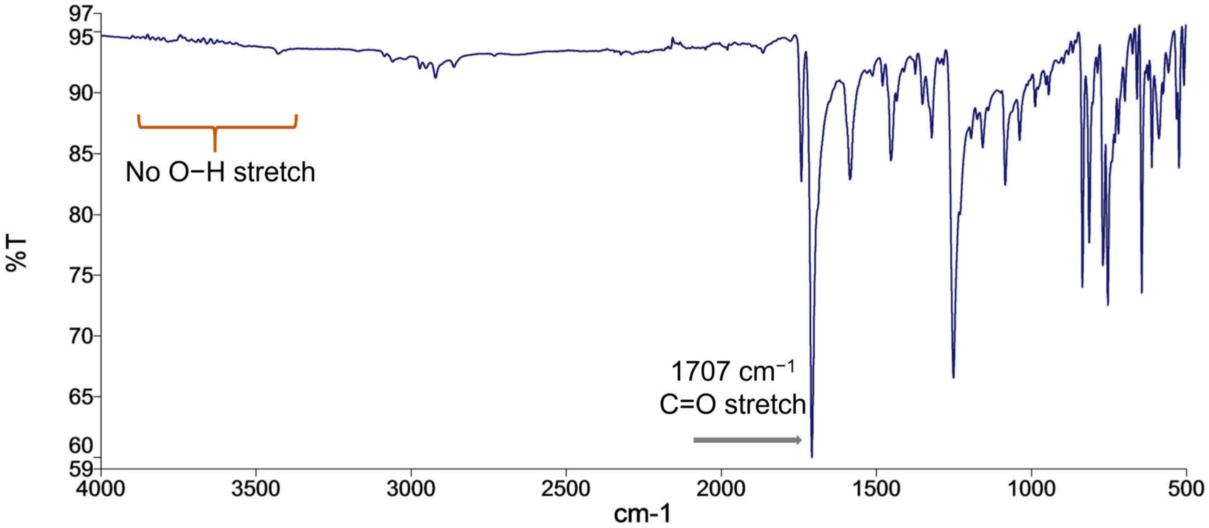

Figure S31. ATR-FT-IR of (±)-**1-O-H₂**.

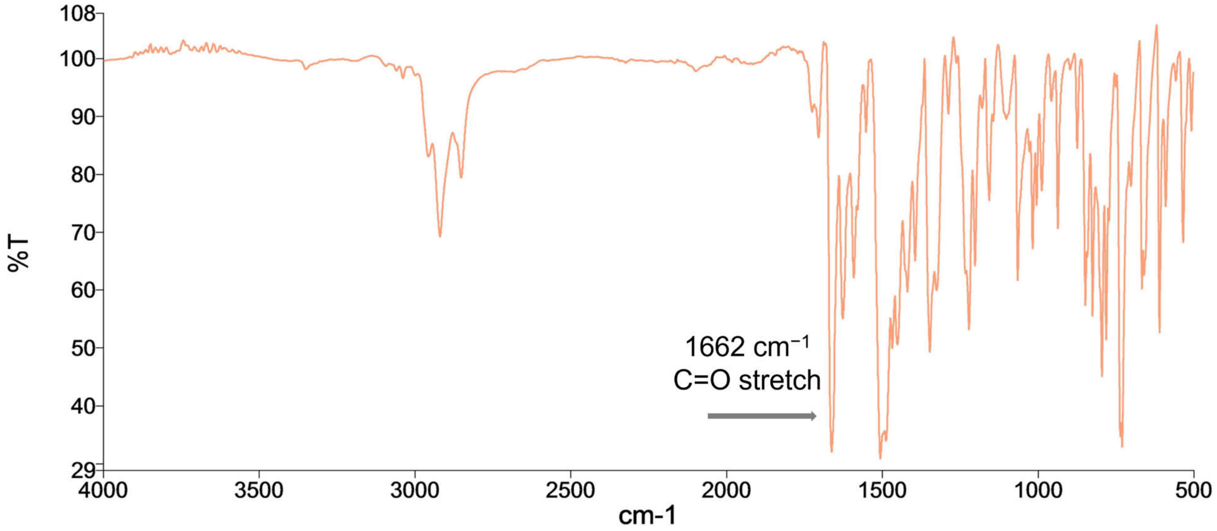

Figure S32. ATR-FT-IR of (±)-**1-C**.

S10. Cartesian Coordinates

Closed closed-shell singlet (1-C_{CS})

B3LYP/def2-SVPP empiricaldispersion=gd3
Charge = 0, Multiplicity = 1, <S^2> = 0
Number of Imaginary Frequencies = 0
Sum of electronic and zero-point Energies = –1913.950934 H
Sum of electronic and thermal Energies = –1913.917702 H
Sum of electronic and enthalpy Energies = –1913.916758 H
Sum of electronic and thermal Free Energies = –1914.015231 H

C	-0.6444	3.3123	-1.1066
C	-0.6326	1.8869	-1.0752
C	0.6854	1.1095	-1.0214
C	1.8225	1.9343	-1.6179
C	1.8092	3.268	-1.6961
C	0.6582	4.0752	-1.208
C	-1.8943	3.9603	-1.1174
C	-3.0811	3.2594	-0.9535
C	-3.0712	1.8729	-0.8404
C	-1.8529	1.1657	-0.9663
C	-4.268	1.187	-0.6066
C	-4.266	-0.1961	-0.4692
C	-3.0816	-0.92	-0.6456
C	-1.8725	-0.2538	-0.9503
C	-3.0917	-2.3036	-0.5084
C	-1.9373	-3.0414	-0.7313
C	-0.7449	-2.4385	-1.1765
C	-0.7083	-1.0141	-1.2488
C	0.4985	-3.2449	-1.4899
C	1.7646	-2.4616	-1.4711
C	1.7823	-1.1321	-1.5967
C	0.5319	-0.2754	-1.7603
C	1.0183	0.8831	0.4748
C	0.3295	-0.0473	-3.2801
C	0.8624	5.3999	-0.8557
C	2.1129	6.2081	-1.0388
C	1.9013	7.5502	-0.5079
C	0.6393	7.6333	0.0279
C	-0.0398	6.3502	-0.1257
C	2.7751	8.6392	-0.4828
C	2.3343	9.8341	0.11

C	1.0425	9.9173	0.6666
C	0.1824	8.8071	0.6313
O	3.1543	5.8593	-1.5682
O	-1.0832	6.0914	0.4493
C	0.5453	-4.5919	-1.8194
C	-0.5675	-5.543	-2.149
C	-0.0053	-6.8595	-2.4349
C	1.3644	-6.7984	-2.358
C	1.7687	-5.4388	-2.0182
C	-0.6614	-8.0458	-2.7705
C	0.1145	-9.1912	-3.0149
C	1.5196	-9.13	-2.9279
C	2.1574	-7.9223	-2.599
O	2.9346	-5.1077	-1.8865
O	-1.7359	-5.273	-2.3684
H	2.7437	1.4512	-1.9192
H	2.7188	3.7125	-2.0611
H	-1.9889	5.0182	-1.2588
H	-4.0146	3.8095	-0.9161
H	-5.2025	1.7248	-0.4956
H	-5.1984	-0.7036	-0.2504
H	-3.9979	-2.8221	-0.216
H	-2.0113	-4.0904	-0.5337
H	2.7356	-2.9235	-1.4461
H	2.7622	-0.6767	-1.669
H	1.1068	1.8566	1.005
H	1.9829	0.3473	0.6004
H	0.2267	0.2923	0.9829
H	-0.5719	0.5688	-3.484
H	1.2047	0.4612	-3.7368
H	0.1995	-1.0197	-3.804
H	3.7687	8.5662	-0.9057
H	2.9917	10.6931	0.1434
H	0.7119	10.84	1.1257
H	-0.8097	8.8632	1.0601
H	-1.7409	-8.0853	-2.8384
H	-0.3692	-10.1243	-3.273
H	2.1105	-10.0162	-3.1195
H	3.2365	-7.8667	-2.537

Open closed-shell singlet (1-O_{CS})

B3LYP/def2-SVPP empiricaldispersion=gd3
Charge = 0, Multiplicity = 1, <S^2> = 0
Number of Imaginary Frequencies = 0
Sum of electronic and zero-point Energies = –1913.892464 H
Sum of electronic and thermal Energies = –1913.858384 H
Sum of electronic and enthalpy Energies = –1913.857440 H
Sum of electronic and thermal Free Energies = –1913.959127 H

C	-0.3389	3.0417	0.0935
C	-0.233	1.6101	0.3514
C	1.0163	1.147	0.8889
C	2.1231	1.9777	0.8861
C	2.0477	3.2914	0.495
C	0.8257	3.9227	0.1792
C	-1.6031	3.5185	-0.2824
C	-2.7345	2.7395	-0.2189
C	-2.6564	1.384	0.037
C	-1.3958	0.7368	0.1417
C	-3.8506	0.6811	0.1792
C	-3.8268	-0.6644	0.4639
C	-2.6292	-1.3716	0.3877
C	-1.4071	-0.7298	0.0523
C	-2.6627	-2.7257	0.6592
C	-1.5394	-3.5074	0.5221
C	-0.363	-3.0372	-0.0796
C	-0.306	-1.6076	-0.3644
C	0.7692	-3.9194	-0.3656
C	1.9085	-3.2908	-0.9132
C	1.9083	-1.9812	-1.3254
C	0.8212	-1.1489	-1.1271
C	1.263	-0.1597	1.6183
C	0.9276	0.155	-1.8947
C	0.8673	5.3204	0.0495
C	2.1066	6.1752	-0.0998
C	1.7271	7.5762	-0.2173
C	0.3666	7.6885	-0.1096
C	-0.2208	6.3671	0.0864
C	2.5388	8.6982	-0.4015
C	1.9269	9.9608	-0.4661
C	0.5287	10.0789	-0.3418
C	-0.2647	8.9343	-0.1574
O	3.2614	5.8054	-0.2335
O	-1.3573	6.2491	0.5104

C	0.843	-5.3148	-0.2179
C	-0.222	-6.368	-0.0214
C	0.4054	-7.6796	0.1045
C	1.7605	-7.5579	-0.0482
C	2.0968	-6.1602	-0.2804
C	-0.1924	-8.9256	0.3128
C	0.6338	-10.0595	0.3854
C	2.0291	-9.9313	0.2415
C	2.6044	-8.6693	0.0195
O	3.2528	-5.7826	-0.3759
O	-1.4187	-6.2698	-0.229
H	3.0771	1.6148	1.2566
H	2.9802	3.8008	0.6131
H	-1.793	4.5006	-0.6069
H	-3.6956	3.2083	-0.4055
H	-4.8069	1.1918	0.1485
H	-4.7632	-1.1713	0.6699
H	-3.575	-3.1903	1.0198
H	-1.6716	-4.4853	0.8811
H	2.8041	-3.8006	-1.1969
H	2.7763	-1.6222	-1.8702
H	0.3542	-0.7408	1.838
H	1.6456	0.0784	2.6343
H	2.0453	-0.7715	1.127
H	1.113	-0.0868	-2.9636
H	1.7881	0.7676	-1.5597
H	-0.0052	0.7379	-1.9409
H	3.6133	8.6007	-0.4877
H	2.5325	10.8467	-0.6064
H	0.0639	11.0552	-0.3869
H	-1.3392	9.0183	-0.0587
H	-1.2654	-9.0176	0.4207
H	0.1963	-11.0354	0.5513
H	2.6601	-10.8089	0.2974
H	3.6751	-8.5642	-0.0996

Open open-shell singlet (1-O_{OS})

UB3LYP/def2-SVPP empiricaldispersion=gd3 guess=mix
Charge = 0, Multiplicity = 1, <S^2> = 0.3475
Number of Imaginary Frequencies = 0
Sum of electronic and zero-point Energies = –1913.913689 H
Sum of electronic and thermal Energies = –1913.879413 H
Sum of electronic and enthalpy Energies = –1913.878469 H
Sum of electronic and thermal Free Energies = –1913.980466 H

C	-0.2282	3.0098	-0.1951
C	-0.107	1.586	-0.4189
C	1.1555	1.1357	-0.96
C	2.2482	2.0039	-1.0202
C	2.1405	3.3271	-0.6556
C	0.9094	3.874	-0.2971
C	-1.4843	3.5404	0.1207
C	-2.6158	2.7596	0.0847
C	-2.5321	1.3854	-0.0965
C	-1.2695	0.7256	-0.1788
C	-3.7278	0.6791	-0.2037
C	-3.7125	-0.6787	-0.4238
C	-2.518	-1.3897	-0.3367
C	-1.283	-0.735	-0.05
C	-2.5761	-2.7633	-0.5315
C	-1.4561	-3.5482	-0.3861
C	-0.2658	-3.0228	0.1301
C	-0.1782	-1.6	0.3744
C	0.8383	-3.8907	0.4115
C	1.9961	-3.3484	0.9672
C	2.0466	-2.0266	1.3489
C	0.9808	-1.1546	1.1142
C	1.4218	-0.1903	-1.6416
C	1.1367	0.17	1.8317
C	0.8947	5.3619	-0.1083
C	1.9853	6.1741	0.4696
C	1.6197	7.5854	0.3782
C	0.3962	7.6942	-0.2509
C	-0.0887	6.3581	-0.5941
C	2.3178	8.7139	0.8095
C	1.7436	9.9768	0.5861
C	0.4961	10.0887	-0.0612
C	-0.1888	8.9386	-0.4888
O	3.0099	5.7458	0.9773
O	-1.0753	6.135	-1.278

C	0.8523	-5.3774	0.2137
C	-0.1978	-6.3758	0.5231
C	0.3341	-7.7096	0.2464
C	1.6433	-7.5975	-0.1756
C	2.0209	-6.1866	-0.1899
C	-0.2835	-8.9547	0.371
C	0.4595	-10.1019	0.0446
C	1.7953	-9.9866	-0.3915
C	2.4	-8.7231	-0.5031
O	3.1141	-5.7558	-0.5219
O	-1.2819	-6.1572	1.0408
H	3.2026	1.6612	-1.4053
H	3.0208	3.9474	-0.7628
H	-1.6407	4.5789	0.3327
H	-3.578	3.2417	0.2222
H	-4.683	1.1926	-0.1903
H	-4.6549	-1.1884	-0.5923
H	-3.505	-3.2416	-0.824
H	-1.5797	-4.5854	-0.6234
H	2.846	-3.9717	1.2131
H	2.9269	-1.6877	1.8846
H	0.5226	-0.7978	-1.8237
H	2.2229	-0.7634	-1.1332
H	1.7851	0.0154	-2.6716
H	1.3268	-0.0377	2.9069
H	0.222	0.7806	1.8652
H	2.0116	0.7409	1.461
H	3.2775	8.6213	1.3012
H	2.2636	10.8688	0.9102
H	0.0638	11.0662	-0.2311
H	-1.1457	9.018	-0.9881
H	-1.3087	-9.0368	0.7081
H	0.004	-11.0799	0.1299
H	2.3594	-10.8763	-0.6391
H	3.4264	-8.6279	-0.8327

Open open-shell triplet (1-O_T)

B3LYP/def2-SVPP empiricaldispersion=gd3
Charge = 0, Multiplicity = 3, $\langle S^2 \rangle$ = 2.0007
Number of Imaginary Frequencies = 0
Sum of electronic and zero-point Energies = –1913.913375 H
Sum of electronic and thermal Energies = –1913.879102 H
Sum of electronic and enthalpy Energies = –1913.878157 H
Sum of electronic and thermal Free Energies = –1913.981104 H

C	-0.2282	3.0098	-0.1951
C	-0.107	1.586	-0.4189
C	1.1555	1.1357	-0.96
C	2.2482	2.0039	-1.0202
C	2.1405	3.3271	-0.6556
C	0.9094	3.874	-0.2971
C	-1.4843	3.5404	0.1207
C	-2.6158	2.7596	0.0847
C	-2.5321	1.3854	-0.0965
C	-1.2695	0.7256	-0.1788
C	-3.7278	0.6791	-0.2037
C	-3.7125	-0.6787	-0.4238
C	-2.518	-1.3897	-0.3367
C	-1.283	-0.735	-0.05
C	-2.5761	-2.7633	-0.5315
C	-1.4561	-3.5482	-0.3861
C	-0.2658	-3.0228	0.1301
C	-0.1782	-1.6	0.3744
C	0.8383	-3.8907	0.4115
C	1.9961	-3.3484	0.9672
C	2.0466	-2.0266	1.3489
C	0.9808	-1.1546	1.1142
C	1.4218	-0.1903	-1.6416
C	1.1367	0.17	1.8317
C	0.8947	5.3619	-0.1083
C	1.9853	6.1741	0.4696
C	1.6197	7.5854	0.3782
C	0.3962	7.6942	-0.2509
C	-0.0887	6.3581	-0.5941
C	2.3178	8.7139	0.8095
C	1.7436	9.9768	0.5861
C	0.4961	10.0887	-0.0612
C	-0.1888	8.9386	-0.4888
O	3.0099	5.7458	0.9773
O	-1.0753	6.135	-1.278

C	0.8523	-5.3774	0.2137
C	-0.1978	-6.3758	0.5231
C	0.3341	-7.7096	0.2464
C	1.6433	-7.5975	-0.1756
C	2.0209	-6.1866	-0.1899
C	-0.2835	-8.9547	0.371
C	0.4595	-10.1019	0.0446
C	1.7953	-9.9866	-0.3915
C	2.4	-8.7231	-0.5031
O	3.1141	-5.7558	-0.5219
O	-1.2819	-6.1572	1.0408
H	3.2026	1.6612	-1.4053
H	3.0208	3.9474	-0.7628
H	-1.6407	4.5789	0.3327
H	-3.578	3.2417	0.2222
H	-4.683	1.1926	-0.1903
H	-4.6549	-1.1884	-0.5923
H	-3.505	-3.2416	-0.824
H	-1.5797	-4.5854	-0.6234
H	2.846	-3.9717	1.2131
H	2.9269	-1.6877	1.8846
H	0.5226	-0.7978	-1.8237
H	2.2229	-0.7634	-1.1332
H	1.7851	0.0154	-2.6716
H	1.3268	-0.0377	2.9069
H	0.222	0.7806	1.8652
H	2.0116	0.7409	1.461
H	3.2775	8.6213	1.3012
H	2.2636	10.8688	0.9102
H	0.0638	11.0662	-0.2311
H	-1.1457	9.018	-0.9881
H	-1.3087	-9.0368	0.7081
H	0.004	-11.0799	0.1299
H	2.3594	-10.8763	-0.6391
H	3.4264	-8.6279	-0.8327

S11. References

[1] G. M. Sheldrick, *SADABS*, **1996**.

[2] G. M. Sheldrick, *Acta Crystallogr. A* **2015**, *71*, 3.

[3] G. M. Sheldrick, *Acta Crytsallogr. C* **2015**, *71*, 3.

[4] C. B. Hübschle, G. M. Sheldrick, B. Dittrich, *J. Appl. Cryst.* **2011**, *44*, 1281.

[5] K. Herb, R. Tschaggelar, G. Denninger, G. Jeschke, *J. Magn. Reson.* **2018**, *289*, 100.

[6] D. C. Harrowven, M. I. Nunn, D. R. Fenwick, *Tetrahedron Lett.* **2002**, *43*, 7345.

[7] P. Ravat, R. Hinkelmann, D. Steinebrunner, A. Prescimone, I. Bodoky, M. Juríček, *Org. Lett.* **2017**, *19*, 3707.

[8] A. V. Astashkin, A. Schweiger, *Chem. Phys. Lett.* **1990**, *174*, 595.

[9] N. Mizuochi, Y. Ohba, S. Yamauchi, *J. Phys. Chem. A* **1997**, *101*, 5966.

[10] N. Mizuochi, Y. Ohba, S. Yamauchi, *J. Phys. Chem. A* **1999**, *103*, 7749.

[11] I. V. Khariushin, P. Thielert, E. Zöllner, M. Mayländer, T. Quintes, S. Richert, A. Vargas Jentzsch, *Nat. Chem.* **2025**, *17*, 493.

[12] S. Grimme, J. Antony, S. Ehrlich, H. Krieg, *J. Chem. Phys.* **2010**, *132*, 154104.

[13] W. J. Hehre, R. Ditchfield, J. A. Pople, *J. Chem. Phys.* **1972**, *56*, 2257.

[14] C. Lee, W. Yang, R. G. Parr, *Phys. Rev. B* **1988**, *37*, 785.

[15] A. D. Becke, *Phys: Rev. A* **1988**, *38*, 3098.

[16] F. Weigend, R. Ahlrichs, *Phys. Chem. Chem. Phys.* **2005**, *7*, 3297.

[17] V. Barone, M. Cossi, *J. Phys. Chem. A* **1998**, *102*, 1995.

[18] T. Koopmans, *Physica* **1934**, *1*, 104.

[19] J. Pommerehne, H. Vestweber, W. Guss, R. F. Mahrt, H. Bässler, M. Porsch, J. Daub, *Adv. Mater.* **1995**, *7*, 551.

[20] K. Günther, N. Grabicki, B. Battistella, L. Grubert, O. Dumele, *J. Am. Chem. Soc.* **2022**, *144*, 8707.
